# Effects of a Community-Based Healthy Lifestyle Intervention Program (Co-HELP) among Adults with Prediabetes in a Developing Country: A Quasi-Experimental Study

**DOI:** 10.1371/journal.pone.0167123

**Published:** 2016-12-09

**Authors:** Norliza Ibrahim, Foong Ming Moy, Intan Attikah Nur Awalludin, Zainudin Mohd Ali, Ikram Shah Ismail

**Affiliations:** 1 Department of Social and Preventive Medicine, University of Malaya, Kuala Lumpur, Malaysia; 2 Julius Centre University of Malaya, Department of Social and Preventive Medicine, University of Malaya, Kuala Lumpur, Malaysia; 3 Department of Dietetic, Hospital Taiping, Perak, Malaysia; 4 State Health Department of Negeri Sembilan, Seremban, Negeri Sembilan, Malaysia; 5 Department of Medicine, University Malaya Medical Centre, Kuala Lumpur, Malaysia; Universita degli Studi di Perugia, ITALY

## Abstract

**Background:**

The prevalence of type 2 diabetes among Malaysian adults has increased by more than two folds over the past two decades. Strategies to collaborate with the existing community partners may become a promising channel for wide-scale dissemination of diabetes prevention in the country. The objectives of this study were to determine the effects of community-based lifestyle interventions delivered to adults with prediabetes and their health-related quality of life as compared to the usual care group.

**Methods:**

This was a quasi-experimental study conducted in two sub-urban communities in Seremban, Malaysia. A total of 268 participants with prediabetes aged between 18 to 65 years old were assigned to either the community-based lifestyle intervention (Co-HELP) (n = 122) or the usual care (n = 146) groups. The Co-HELP program was delivered in partnership with the existing community volunteers to incorporate diet, physical activity, and behaviour modification strategies. Participants in the Co-HELP group received twelve group-based sessions and two individual counselling to reinforce behavioural change. Participants in the usual care group received standard health education from primary health providers in the clinic setting. Primary outcomes were fasting blood glucose, 2-hour plasma glucose, and HbA1C. Secondary outcomes included weight, BMI, waist circumference, total cholesterol, triglyceride, LDL cholesterol, HDL cholesterol, systolic and diastolic blood pressure, physical activity, diet, and health-related quality of life (HRQOL).

**Results:**

An intention-to-treat analysis of between-groups at 12-month (mean difference, 95% CI) revealed that the Co-HELP participants’ mean fasting plasma glucose reduced by -0.40 mmol/l (-0.51 to -0.28, p<0.001), 2-hour post glucose by -0.58 mmol/l (-0.91 to -0.24, p<0.001), HbA1C by -0.24% (-0.34 to -0.15, p<0.001), diastolic blood pressure by -2.63 mmHg (-3.79 to -1.48, p<0.01), and waist circumference by -2.44 cm (-4.75 to -0.12, p<0.05) whereas HDL cholesterol increased by 0.12 mmol/l (0.05 to 0.13, p<0.01), compared to the usual care group. Significant improvements were also found in HRQOL for both physical component (PCS) by 6.51 points (5.21 to 7.80, p<0.001) and mental component (MCS) by 7.79 points (6.44 to 9.14, p<0.001). Greater proportion of participants from the Co-HELP group met the clinical recommended target of 5% or more weight loss from the initial weight (24.6% vs 3.4%, p<0.001) and physical activity of >600 METS/min/wk (60.7% vs 32.2%, p<0.001) compared to the usual care group.

**Conclusions:**

This study provides evidence that a culturally adapted diabetes prevention program can be implemented in the community setting, with reduction of several diabetes risk factors and improvement of HRQOL. Collaboration with existing community partners demonstrated a promising channel for the wide-scale dissemination of diabetes prevention at the community level. Further studies are required to determine whether similar outcomes could be achieved in communities with different socioeconomic backgrounds and geographical areas.

**Trial Registration:**

IRCT201104106163N1

## Introduction

Type 2 diabetes is a global public health problem and has become common among the developing countries. According to the International Diabetes Federation [1], 8.3% or 382 million of adults around the world are affected by diabetes. It is predicted that people with diabetes will increase to 592 million in 2035. Once a disease of the West, type 2 diabetes has now spread to every country in the world. Type 2 diabetes poses a huge economic burden and developing countries bear the highest burden as 80% of cases occur in these countries. Asian countries account for 60% of the world’s diabetes population and is expected to increase further in the next several decades. Rapid urbanization, nutritional transition, and sedentary lifestyles have contributed to the accelerated increase of diabetes epidemic in this region. Similarly, this rapid transition has also lead to an increase of prediabetes and diabetes prevalence in Malaysia, which is a middle income country experiencing rapid economic growth and urbanization. Even more worrying is the fact that almost half of the population with diabetes in Malaysia were unaware that they already have the disease [2]. The prevalence of diabetes has increased more than double from 1996 to 2011. This has placed Malaysia at number ten among countries with the highest diabetes prevalence globally[3]. These realities exist despite the implementation of large scale health campaigns to enhance diabetes awareness in the community. It was predicted that the diabetic population in Malaysia will continue to rise from 2.6 million (15.2%) in 2011 to 4.5 million (21.6%) by the year 2020. In addition, the increasing prevalence of overweight and obesity in all segments of population has caused the burden of prediabetes and diabetes to continue to escalate [4]. Identifying strategies to prevent diabetes is indeed a public health priority.

Prediabetes is a condition in which individuals have blood glucose levels higher than normal but not high enough to be classified as diabetes[5]. People with prediabetes are at high risk of developing diabetes[6, 7] and cardiovascular diseases [8, 9]. A meta-analysis of prospective cohort studies showed that the annual incidence of type 2 diabetes was 4.6% for those with impaired fasting glucose (IFG), 6.3% for those with impaired glucose tolerance (IGT), and 12.1% for those with both IFG and IGT [10]. Not all individuals with prediabetes progress at the same rate, but approximately 37% of them would develop diabetes in four years if they receive no intervention[11]. Primary prevention is possible by modifying the risk factors of diabetes. Evidence from large scale clinical trials [11–13]demonstrated a 58% reduction of diabetes incidence through lifestyle modification. Although compelling evidence exists, it has yet to be widely adopted at the community setting. Translating the evidence to public health is a challenge, and there is a clear need to balance effectiveness and adaptability to the community setting [14]. More recent evidence showed that lifestyle intervention can be translated in diverse community settings such as Diabetes Care Centre[15], Young Men's Christian Association (YMCA)[16] and churches[17, 18]. Ali et al.[19] conducted a systematic review and meta-analysis on twenty eight studies that translated the Diabetes Prevention Program (DPP) weight loss intervention. They found a clinically significant (4 to 5%) weight loss at 12 months follow up regardless of types of personnel (i.e., medical and allied health professionals, lay educators from the community) and electronic media-assisted methods delivering the program. They concluded that the change in weight was similar regardless of whether the intervention was delivered by clinically trained professionals or lay educators. Another systematic review and meta-analysis by Dunkley et al.[20] concluded that pragmatic interventions were effective and adherence to guidelines’ recommendation was significantly associated with greater weight loss. However, studies from developing countries on pragmatic diabetes prevention programs are scarcely documented. Strategies to collaborate with existing community partners may be a promising channel for wide-scale dissemination of a low-cost approach to diabetes prevention in our country [16]. To date only one study in Malaysia has reported the effectiveness of lifestyle intervention among prediabetes patients conducted in primary care setting [21], while others were mainly observational studies and prevalence studies of diabetes in the population [22–28]. Furthermore, little is known about whether community-based lifestyle intervention can produce better health-related quality of life for prediabetes patients [29]. We hypothesized that a community-based approach to lifestyle modification is more effective than usual care for patients with prediabetes. Therefore, the aims of this study were to determine the effects of community-based lifestyle modification intervention among adults with prediabetes and their health-related quality of life as compared to the usual care group.

## Materials and Methods

The methods of this study adhered to the Transparent Reporting of Evaluations with Nonrandomized Designs (TREND) statement [30, 31]. S1 TREND checklist, S1 Study protocol, S1 Fig, S1 and S2 Tables are available as supporting information.

### Study design and setting

This was a quasi-experimental study with repeated measures, conducted in two sub-urban communities in Seremban, Negeri Sembilan, Malaysia. Based on the 2010 reports [32–34], two communities i.e., Ampangan and Senawang with similar demographic and socioeconomic characteristics were selected. The study participants were allocated to the intervention or control group depending on which community they were enrolled at the time of the baseline screening. This study involved collaboration between the research team from the Department of Social and Preventive Medicine, University of Malaya and community volunteers from the Malaysian Diabetes Association (MDA), local residential committees and primary health clinics. The study was conducted in two phases. The first phase involved planning, preparation of intervention modules, setting up collaboration with various stakeholders, training of community volunteers, and recruitment of participants. The second phase of the study involved implementation of intervention, data collection, follow-up, and data analyses (S1 Fig).

The community-based healthy lifestyle intervention program (Co-HELP) was designed following review of evidences and guidelines [11–13, 15–17, 21, 35–39]. The group-based program was adopted from publicly available materials from the US Group Lifestyle Balance of Diabetes Prevention Program (DPP Research Group) [40], combined with materials from the Integrated Educational Module for Non-Communicable Diseases from the Ministry of Health Malaysia[41]. Modification was tailored according to the needs and cultural sensitivity of the community. Few consultations were conducted with stakeholders (i.e., dieticians, physical trainers, family medicine specialists, local community organizations and potential participants) prior to the intervention. The education materials were refined and pre-tested on the community-volunteers prior to delivery. The content was based on the Health Belief Model as its theoretical framework[42].

### Eligibility criteria

Participants were eligible if they lived or worked within the selected communities, aged between 18–65 years old, able to read and understand the Malay language (Bahasa Malaysia) or English language, were at risk of type 2 diabetes with fasting blood glucose concentration between 5.6 to 6.9 mmol/L, and/ or 2-hour 75 g oral glucose tolerance test concentration between 7.8 mmol/L and 11.0 mmol/L [43], body mass index (BMI) between 23–39 kg/m^2^ and had no contraindications to participate in weight management program or physical activity. The exclusion criteria included clinical history of diabetes or newly diagnosed diabetes at the time of screening, involved in other weight management program, took any herbal medications to reduce weight, had clinical history of cardiovascular diseases occurred within the past six months, had advanced arthritis, uncontrolled hypertension, any form of cancers that require treatment, pregnant and other causes (i.e., psychological disorder or physically disabled) which could interfere with participation.

### Methods of recruitment

The participants were recruited from the general population through healthcare providers and presentations at community-halls, mosques, as well as through media exposure like radio and Malaysian Diabetes Association’s website, brochures, banners and posters. Between October 2011 and January 2012, eight health affairs were conducted in the community to screen for eligible participants. The screening process was based on the guidelines by the Ministry of Health, Malaysia [38]. The Physical Activity Readiness Questionnaire (PAR-Q) was used to identify participants with contraindication to exercise. They were referred to a family medicine specialist for medical clearance prior to participation.

### Interventions

Intervention of the participants commenced in January, 2012 and ended in June, 2013. The 12 months intervention program consisted of two parts; with first six months of active period and next six months of maintenance period. In total, participants in the intervention group received twelve group-based sessions of 90 minutes each and minimum of two individual counselling sessions with the dietician and researcher to reinforce behavioural change. Most of the group sessions were held during weekends, at community hall or recreational park. The structured group sessions in the first six months (active period) were conducted biweekly for the first three months (Sessions 1–6) and monthly in the next three months (Sessions 7–9). During the maintenance period, the group sessions were held monthly for three months (Session 10–12) and participants were followed up through telephone calls or home visits for the last three months. All group sessions were conducted either in large group of 20 people or smaller group of five to eight people depending on the type of activities (i.e. lecture, seminar, group work or discussion). Table 1 provides detailed information on session titles, frequency, content focus and the Health Belief Model constructs.

**Table 1:** The session titles, health belief model construct, content, and purposes of the Community-based Healthy Lifestyle Intervention Program (Co-HELP).

Health Belief Model	Session and title	Content and purpose of the session
Perceived susceptibility.	**Session 1**: Introduction to Co-HELP and risk factors for type 2 diabetes.	• Introduction to the program and other group members.
		• To increase knowledge about diabetes and the risk factors.
		• To understand the signs, symptoms, complications and management of chronic diseases.
		• Emphasis on benefits of lifestyle modification; diet, physical activity, and smoking and alcohol cessation.
Perceived benefits.	**Session 2**: Healthy eating.	• To increase knowledge about healthy eating, principles of healthy diet, food pyramid, and portion size.
		• Improving quality of food choices with tips to reduce salt, sugar, and fatty intake.
		• What is the “healthy plate model” and “snacks food”.
		• Diseases related to unhealthy diet.
		• Pre-and Post-test questionnaire.
Perceived benefits.	**Session 3**: Guide to exercise and physical activity.	• Difference between exercise and physical activity.
		• Concept of energy balance, diet and exercise.
		• Preparation for exercise: types and different phases of exercise.
		• For participants to know the appropriate types of exercise for middle aged people and weight reduction purposes.
		• Common barriers to exercise and coping strategies.
		• Counting the heart rate and goal setting.
Perceived barriers	**Session 4**: Keys to healthy eating and groceries shopping.	• Identify obstacles; learn about problem solving and decision making.
		• Tips to improve the quality of food choices and shopping tips i.e. reading food labels and nutritional facts, preparation of the shopping lists and planning ahead.
		• To know what types of food are rich in salt, sugar, and fats?
		• What are artificial sweeteners?
		• Tips on menu choices while eating outside i.e. restaurants or food stalls.
		• Good or bad choice of specific dishes according to different ethnic groups.
Perceived barriers.	**Session 5**: Guide to lose weight and healthy cooking methods.	• Introduction to overweight and obesity problems.
		• Measuring your own weight, BMI, waist circumference, BP and blood sugar.
		• Knowing your ideal body weight and setting goals for weight reduction.
		• Health benefits of achieving the targeted goals and energy balance.
Cues to action.	**Session 6**: Exercise: 10,000 steps.	• Outdoor activity at the recreational park.
		• Group walking in the community.
Cues to action.	**Session 7**: Dietary interactive session.	• Interactive session with participants, divided in smaller groups of 5–8 people.
		• Problem solving, role play and group presentations.
		• To recap and give feedback on previous dietary modules.
Cues to action.	**Session 8**: Indoor exercise and during leisure time.	• Interactive session with participants.
		• Flexibility and resistance exercise using elastic bands.
		• Aerobic exercise.
Cues to action.	**Session 9**: Creating a healthier recipe, cooking demonstration and meal contest.	• Interactive session with participants.
		• Practical meal planning and preparation.
		• How to modify two famous local recipes of Negeri Sembilan into healthier recipes.
		• Cooking demonstration by community-volunteers.
		• Tips to reduce consumption of saturated fat (especially from coconut milk) in local dishes.
		• Conducted a “healthy meal contest” by modification of recipes.
Self-efficacy.	**Session 10**: Strengthen you exercise programs.	• Outdoor aerobic exercise.
		• Keep active and prevent injuries.
		• How to achieve 150 minutes per week and moderate intensity exercise.
Self-efficacy.	**Session 11**: Motivational talk and sharing of experience.	• How to maintain your weight during Ramadhan (fasting month) and Syawal (Eid celebration).
		• Sharing of personal experiences by two role models (i.e., a retired government servant and a diabetic patient).
		• Encourage other participants to share their own experiences and motivations about staying healthy.
Self-efficacy.	**Session 12**: Looking back and looking forward.	• Looking backward is to flash back on all the activities that have been conducted.
		• Looking forward is how to keep motivated and sustained healthy lifestyle.
		• All participants signed up a “My personal Lifestyle Contract”.
		• Token of appreciation to all participants
		• Selection of best male and best female participants who achieved the most weight loss, full attendance in all activities and managed to return to the normal blood glucose level.

The research team prepared study materials, support and supervised the lifestyle intervention sessions, as well as provided training and advocacy to local leaders and community volunteers. Group sessions were coordinated and facilitated by trained community volunteers with supervision from the researchers. To ensure the fidelity of the intervention, the community volunteers were trained in a two-day training workshop prior to the delivery of intervention. The community volunteers were also involved with the participants’ anthropometry measurements, monitoring of attendances and setting up arrangement for home-visits. Home-visits were conducted especially for those who missed their follow ups or could not be reached by telephone calls. The community volunteers consulted the researchers whenever they encountered problems during the intervention sessions. The community-based healthy lifestyle intervention incorporated diet, physical activity and behaviour modification strategies. The goals set for lifestyle intervention were based on the recommendations by the Ministry of Health Malaysia [38], to produce modest but achievable outcomes mainly reduction of 5–10% of initial body weight for all overweight and obese participants, reduction of calorie intake (20–25 kcal/kg body weight) and, an increase from light to moderate physical activity (≥ 600 METs-minute/week).

#### Diet

The dietary advice given by the dietician was based on the Malaysian Nutritional Guidelines [44, 45]. Each participant’s daily recommended energy requirement was estimated by the dietician based on their 3-day food records. Information gathered during the initial appointment with dietician was used to design the participants’ diet plan, short term and long term weight loss goals and behaviour modification. The participants were encouraged to aim for5-10% weight loss at 6-month and continue to lose weight up to 12 months.

#### Physical Activity

Participants were encouraged to increase their physical activity to a minimum of 150 minutes per week and aimed for moderate intensity exercises. A six-kilometre walking track was also set up within the community. The pathway chosen for walking activity was away from the main road to ensure safety of the participants. At every kilometre, a banner was put up with motivational health related messages. Participants were encouraged to form their own walking groups and use pedometers to assess their progress.

#### Self-monitoring

Participants were also encouraged to self-monitor their body weight, biochemical results, diet, and physical activity in a specially designed diary. In addition, they also received hand-outs, pamphlets, and booklets on various health issues. Pre-tests and post-tests were conducted after each educational session to evaluate their understanding of the topics discussed earlier.

#### Control group

Participants in the usual care group received standard health education from primary care providers in the clinic. In addition, they were also provided with pamphlets and booklets about various health topics. The same diary was also given to them to record their weights, diets, physical activities and other blood test results.

### Measurements

#### Anthropometry and blood pressure

Anthropometric measurements, which included weight, height and waist circumference, and blood pressure, were taken by trained personnel, using calibrated measuring tools. The participants were instructed to wear light clothing and without shoes during measurements. Height and weight were taken using a stadiometer and SECA digital scale, to the nearest 0.1 cm and 0.1 kg, respectively. BMI was calculated using the formula of weight in kg divided by height in m^2^ (kg/m^2^). Using the revised BMI cut-offs for Asian classification [46], participants with BMI between 23.0 kg/m^2^ to <27.5 kg/m^2^wereclassified as overweight and a BMI of ≥ 27.5 kg/m^2^ was classified as obese. Waist circumference was taken to the nearest 0.1 cm using SECA measuring tape and according to the WHO procedures (1995) [47]. Blood pressure was measured with an automatic digital blood pressure monitor (OMRON HEM -907 model). The participants were required to be seated with two measurements taken for an average value, after resting for at least 10 to 15 minutes.

#### Biochemical

All participants were required to fast overnight for a minimum of eight hours prior to each blood test. Plasma levels of fasting glucose and 2-hour post 75 g glucose load were assayed using the GLUC method for the Dimension Clinical Chemistry System (by Siemens, Newark, U.S.A). Participants were advised not to engage in strenuous physical activity for at least 12 hours before blood sampling in order to minimize the influence of any acute incidental reaction. The Bio-Rad D-10 Haemoglobin A1C program system was used for the percentage determination of % HbA1C using the ion-exchange high performance liquid chromatography method (HPLC). The total cholesterol (TC), high density lipoprotein (HDL) and triglyceride (TG) were analyzed using the Dimension Clinical Chemistry System (by Siemens, Newark, U.S.A.). The low density lipoprotein (LDL) was calculated using the Friedwald equation [48].

#### Physical activity

Physical activity (PA) was measured using the short form of the International Physical Activity Questionnaire (IPAQ). The IPAQ was translated and validated in different languages including the Malay language[49]. Participants’ weekly MET-minutes of walking, moderate and high intensity activities were summed up and categorized into low physical activity (i.e., < 600 MET-minutes/week), moderate physical activity (i.e., 600–1500 MET-minutes/week), and high physical activity (i.e., ≥ 1500 MET-minutes/week) [50].

#### Dietary intake

The dietary intake was measured using the 24 hours diet recall. Participants’ were asked to record their dietary intake for three days (two week-days and one week-end) and the average measurement was taken.

#### Health-related quality of life

Measures of health-related quality of life (HRQOL) describe how people perceive their health and function across physical, mental and social well-being during their usual daily activities. HRQOL is now more often used to evaluate the effects of health promotion programs and treatment outcomes. The HRQOL of our participants was assessed using a bilingual version of short-form health survey (SF-36) questionnaire. The SF-36 was translated and validated in Malaysia [51] and the Malay version of the SF-36 has shown to be reliable and valid whereby the Cronbach alpha coefficients for all subscales exceeded the recommended 0.70 [52]. It contains 36 items, which measures eight health domains, namely; physical functioning (PF), role-physical (RF), bodily pain (BP), general health (GH), vitality (VT), social functioning (SF), role-emotion (RE) and mental health (MH)[53]. The eight domains were scored from 0 to 100 indicating worst to best possible health. All the scores were further summarized into the Physical Component Summary score (PCS) and Mental Component Summary score (MCS).

### Outcome measures

The primary outcome measures for this study were fasting blood glucose, 2-hour plasma glucose, and HbA1C. Secondary outcome measures included weight, BMI, waist circumference, total cholesterol, triglyceride, LDL cholesterol, HDL cholesterol, systolic and diastolic blood pressure, physical activity, dietary intake and HRQOL. All these measurements were taken at baseline, 6- and 12-month follow-up except for HRQOL that was measured at baseline and 12-month. The primary assessment time-point of this study was at 6-month to measure the immediate effectiveness of the intervention. The final visit, which occurred at 12-month was used to measure the sustainability of the intervention.

### Sample size

The sample size calculation was based on the results by Kulzer et al. [54]. The PREDIAS revealed significant differences for weight, fasting blood glucose, and physical activity. The values were entered into the STATA program to calculate the sample size for the two-group trial with a power of 80% and significant level preset at 0.05, to detect a difference of 2.4 kg weight loss, 6.1 mg/dL or 0.03 mmol/L in fasting blood glucose and 28.7 minutes per week in physical activity. Assuming a drop-out rate of 30% and to ensure adequate power was established for most of the outcome measures, the total number of participants required for this study was 244 (122 for each group).

### Statistical analyses

Participants’ demographic characteristics, anthropometric measurements and clinical indicators were compared between groups using independent t-test for normally distributed variables and Mann Whitney test for non-normally distributed variables. Chi-square test was used for categorical variables. The analysis was performed according to the intention-to-treat (ITT) principle. The missing values were replaced by carrying forward the last readings. Repeated measures ANOVA was conducted to examine the changes overtime within and between-groups. The baseline value of lipid profiles, blood pressure, co-morbidities, and medication used were used as covariates in the analysis for lipid profiles and blood pressure measurements. The results of the outcomes from baseline to follow ups at 6- and 12-month were presented as mean group differences with 95% confidence interval. Comparison of intervention groups across domains of HRQOL scores was carried out using the analysis of covariance (ANCOVA) with Bonferroni correction. Two-tailed p values of less than 0.05 were considered statistically significant. The effect size was based on eta square as proposed by Cohen (1998): 0.01 = small effect, 0.06 = moderate effect, and 0.14 = large effect [55]. A sensitivity analysis using complete cases was performed to test the attrition or imputation bias. Statistical package for the social sciences (SPSS) software version 19 was used for all analyses.

### Ethical approval and consent

The Medical Ethics Committee of University Malaya’s Medical Centre (MEC ref. no. 841.3) approved the study protocol. The study also received permission from the State Health Department of Negeri Sembilan. Written informed consent was obtained from all participants before the study began.

## Results

### Recruitment and response rates

**Fig 1:**
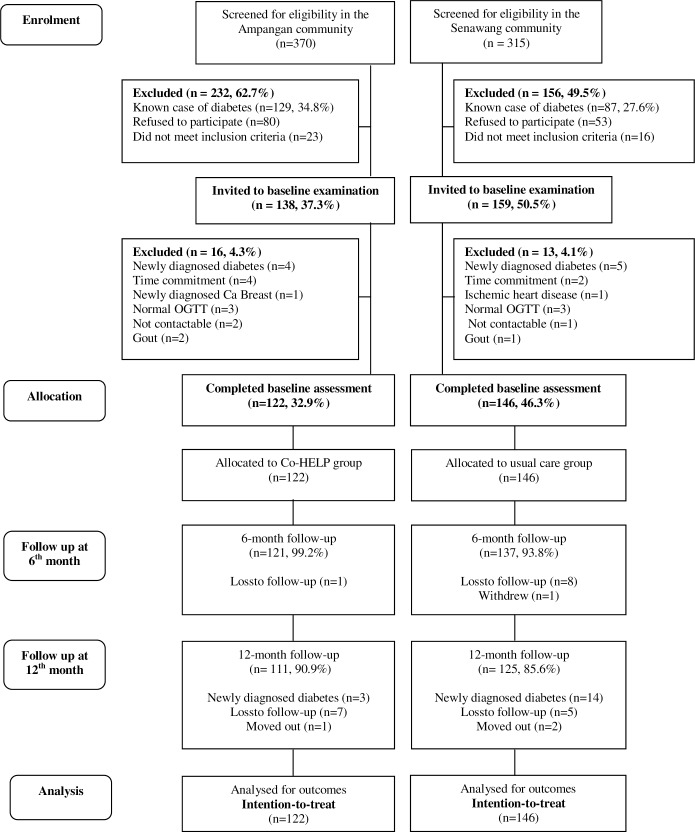
Study flow of participants.

Fig 1 illustrates the flow of participants through each stage of the study; enrolment, allocation, exposure to intervention, and follow-ups. Of the 685 individuals screened, 370 individuals (54.1%) were from the Ampangan site and 315 individuals (45.9%) from the Senawang site. Of the 370 individuals screened at the Ampangan site, 232 individuals (62.7%) were excluded due to reasons including known case of diabetes (129), refusal to participate (80), and did not meet the inclusion criteria (23). One hundred thirty-eight individuals (37.3%) were potentially eligible and were invited for the baseline examination. Of the 138 individuals, 16 individuals (4.3%) were excluded due to reasons such as newly diagnosed diabetes (4), time commitment (4), newly diagnosed breast cancer (1), normal OGTT (3), not contactable (2) and diagnosed with gout (2). On the other hand, of the 315 individuals screened in Senawang, 156 individuals (49.5%) were excluded due to known case of diabetes (87), refusal to participate (53) and did not meet the inclusion criteria (16). A total of 159 individuals (50.5%) were potentially eligible and were invited for baseline examination. Of the 159 individuals, 13 individuals (4.1%) were excluded due to reasons including newly diagnosed diabetes (5), time commitment (2), ischemic heart disease (1), normal OGTT (3), gout (1), and not contactable (1). Finally, 122 and 146 eligible individuals from the Ampangan and Senawang sites respectively consented to the study. Participants from the Ampangan site were allocated to the Co-HELP group while the participants from the Senawang site were assigned to the usual care group.

### Adherence to the intervention

On average, intervention participants attended 80% (range 55% - 95%) of the group sessions in the first 6 months. Seventy participants (70/122, 58%) attended all 12 sessions during the 12 months of intervention.

#### Trial profile

Thirty-two participants in the intervention (n = 11) and usual care (n = 22) groups did not complete the study over the 12 months period. Reasons for not completing were loss to follow-up, withdrew from the study, moved out, and newly diagnosed diabetes. There was no significance difference in baseline characteristics among those who default the study. The 12-month assessment was completed by 111 participants (90.9%) from the Co-HELP group and 125 participants (85.6%) fromthe usual care group. Over the course of study, 3 participants (2.4%) from the Co-HELPgroup and 14 participants (9.6%) from the usual care group developed type 2 diabetes (Fig 1).

### Baseline characteristics of the participants

The baseline characteristics of the participants are presented in Tables 2 and 3. The mean (SD) age of the participants was 52.7(7.6) years with 63.1% being females, married (90.7%) and of the Malay ethnicity (89.2%). Most participants had at least secondary school education and of low to moderate household income. More than half of them had family history of diabetes (53.7%) and had cardiovascular risk factors, mainly hypertension and dyslipidemia. About 17.0% of them were smokers, 12.0% consumed alcohol, and 64.6% were physically inactive. The mean (SD) BMI was in the obese range of 30.1(4.8) kg/m^2^. Based on the glycemic indicators; 29.1% had IFG, 8.2% had IGT and 62.7% had both. There were no significant differences between the Co-HELP and usual care groups in baseline characteristics (p>0.05), except more participants in the usual care group had hypertension and were on antihypertensive medication. However, there were no significant differences in the systolic and diastolic blood pressure measurements between both groups.

**Table 2:** Baseline socio-demographics and health characteristics of participants.

Characteristics	Co-HELP(n = 122)	Usual care(n = 146)	p-valueª
Age, years; mean(SD)	53.3(7.1)	52.2(8.1)	0.255
Gender; n(%)			
Male	41(33.6)	55(37.7)	0.489
Female	81(66.4)	91(62.3)	
Ethnicity; n(%)			
Malay	110(90.2)	129(88.4)	0.750
Chinese	7(5.7)	8(5.5)	
Indian	5(4.1)	9(6.2)	
Level of education; n(%)			
Primary	21(17.2)	29(19.9)	0.850
Secondary	81(66.4)	93(63.7)	
Tertiary	20(16.4)	24(16.4)	
Marital status; n(%)			
Married	114(93.4)	129(88.4)	0.154
Unmarried/divorced/widowed	8(6.6)	17(11.6)	
Monthly household income; n(%)			
Low	60(49.2)	63(43.2)	0.403
Moderate	38(31.1)	57(39.0)	
High	24(19.7)	26(17.8)	
Co-morbidities; n(%)			
Hypertension	94(77.0)	131(89.7)	0.005
Dyslipidemia	49(40.2)	49(33.6)	0.264
Health behaviour; n(%)			
Current smoker, yes	7(5.7)	10(6.8)	0.909
Alcohol use, yes	5(4.1)	7(4.8)	0.933
Regular exercise >3 times per week, yes	22(18.0)	24(16.4)	0.730
Medications in current use; n(%)			
Anti-hypertensive	94(77.0)	131(89.7)	0.005
Lipid lowering	49(40.2)	49(33.6)	0.264
Family history of type 2 diabetes; n(%)	59(48.4)	85(58.2)	0.107

Values are n (%) except for age in mean (SD).

ª Determined with χ^2^ test for categorical variables and independent t-test for continuous variables and, significant at p-value <0.05. Low income: <1,500 MYR, moderate income: 1,500–3,000 MYR, high income: > 3,001 MYR. MYR: Malaysian Ringgit. Classification from the Department of Statistics Malaysia (currency conversion: 1.00 MYR = USD 0.34).

**Table 3:** Baseline clinical, physical activity, diet, and health-related quality of participants.

Variable	Co-HELP (n = 122)	Usual care (n = 146)	p-value^a^
Weight, kg; mean(SD)	75.30(14.07)	75.50(13.15)	0.903
Body Mass Index(BMI), kg/m^2^; mean(SD)	30.08(4.99)	30.10(4.58)	0.974
BMI categories; n(%)			
Overweight (23.0–27.4 kg/m^2^)	33(27.1)	41(28.1)	0.552
Obese (≥ 27.5 kg/m^2^)	89(72.9)	105(71.9)	
Waist circumference, cm; mean(SD)	93.86(8.93)	95.06(9.92)	0.303
Fasting Plasma Glucose, mmol/l; mean(SD)	6.16(0.58)	6.19(0.46)	0.638
2-h Post-load Glucose, mmol/l; mean(SD)	8.44(1.68)	8.66(1.41)	0.241
HbA1c,%; mean(SD)	6.11(0.48)	6.18(0.43)	0.189
Diagnosis of Prediabetes; n(%)			
IFG	36(29.5)	42(28.8)	0.071
IGT	15(12.3)	7(4.8)	
Both	71(58.2)	97(66.4)	
Systolic blood pressure, mmHg; mean(IQR)	136.00(10.00)	139.00(19.25)	0.964
Diastolic blood pressure, mmHg; mean(IQR)	80.00(7.00)	84.00(8.00)	0.050
Total cholesterol, mmol/l; mean(SD)	5.26(0.84)	5.25(0.91)	0.971
Triglyceride, mmol/l; median(IQR)	1.30(0.90)	1.53(0.84)	0.645
HDL cholesterol, mmol/l; mean(SD)	1.19(0.28)	1.17(0.33)	0.488
LDL cholesterol, mmol/l; mean(SD)	3.32(0.83)	3.44(0.86)	0.286
Physical activity, total MET-mins/wk; mean(SD)	652.49(352.77)	650.88(595.45)	0.979
Dietary, total energy kCal/day; mean(SD)	1479.66(287.88)	1498.62(298.41)	0.599
HRQOL, SF-36 scales, mean(SD)			
Physical functioning (PF)	80.52(18.51)	83.08(14.77)	0.210
Role physical (RP)	84.46(19.82)	87.52(18.62)	0.194
Bodily pain (BP)	83.93(15.95)	84.71(18.03)	0.708
General health (GH)	72.49(16.05)	68.82(18.24)	0.085
Vitality (VT)	74.79(13.53)	74.05(13.26)	0.654
Social functioning (SF)	91.04(15.08)	92.29(15.76)	0.497
Role emotion (RE)	88.41(18.04)	86.35(19.02)	0.368
Mental health (MH)	83.28(11.53)	80.96(12.54)	0.119
Physical Component Score (PCS)	40.41(6.62)	40.58(6.58)	0.837
Mental Component Score (MCS)	42.18(5.72)	41.70(5.82)	0.501

Values are mean (SD) or median (IQR) where appropriate.

^a^Determined with independent t-test or Mann Whitney test for continuous variables, significant at p<0.05. IFG: impaired fasting glucose, IGT: impaired glucose tolerance, BMI categories based on revised cut-offs for Asian classification.

### Main outcomes

#### Effects of Co-HELP on glucose metabolism

An intention-to-treat analysis of between groups at 12-month (mean difference, 95% CI) revealed that the Co-HELP participants had greater decreased in the FPG -0.40 mmol/l (-0.51 to -0.28), 2-hPG -0.58 mmol/l (-0.91 to -0.24) and HbA1C -0.24% (-0.34 to -0.15), with all p-values <0.001 (Table 4). The FPG in the Co-HELP group significantly decreased at 6- and 12-month follow-up by -0.59 mmol/l and -0.47 mmol/l respectively, p<0.001 for both. In contrast, the usual care group also showed a decrease in FPG at 6-month (-0.03 mmol/l) but was not sustained at 12-month follow up (+0.07 mmol/l, p>0.05). Relative to the usual care group, the Co-HELP group demonstrated substantial decrease in 2-hPG by -0.49 mmol/l and -0.59 mmol/l at 6- and 12-month respectively (p<0.001 for both). The usual care group had an increase of 0.15 mmol/l at 6- and 12-month follow-ups (p>0.05). Similar pattern of changes were observed for HbA1C whereby the Co-HELP group demonstrated a decrease of -0.15%and -0.13% (p<0.001) as compared to an increase of 0.10%and 0.14% (p>0.05) for the usual care group at 6- and 12-month follow-up respectively. Sensitivity analysis using complete cases were carried out and the results were consistent with the primary analysis (S1 and S2 Tables). The treatment effects on most of the outcome measurements including HRQOL were comparable with the intention-to-treat analysis.

**Table 4:** Changes in outcomes from baseline to 6- and 12-month follow-up with differences in within and between groups over time.

Outcome	Co-HELP (n = 122)		Usual care (n = 146)		Mean difference (95% CI)between groups ^b^ x time		
	Mean change (95% CI) from baseline		Mean change (95% CI) from baseline				
	6-month	12-month	6-month	12-month	6-month	12-month	partial η^2^
FPG (mmol/l)	-0.59(-0.75 to -0.44)^a^	-0.47(-0.60 to -0.33)^a^	0.03(-0.14 to 0.08)	0.07(-0.05 to 0.19)	-0.31(-0.39 to -0.24)^b^	-0.40(-0.51 to -0.28)^b^	0.151
2-hPG (mmol/l)	-0.49(-0.79 to -0.19)^a^	-0.58(-0.90 to -0.27)^a^	0.15(-0.01 to 0.31)	0.15(-0.01 to 0.31)	-0.44(-0.77 to -0.11)^b^	-0.58(-0.91 to -0.24)^b^	0.042
HbA1C (%)	-0.15(-0.23 to -0.06)^a^	-0.13(-0.22 to -0.05)^a^	0.10(0.03 to 0.17)^a^	0.14(0.07 to 0.20)^a^	-0.18(-0.27 to -0.09)^b^	-0.25(-0.35 to -0.15)^b^	0.085
Weight (kg)	-1.99(-2.69 to -1.28)^a^	-2.12(-2.83 to -1.40)^a^	0.08(-0.28 to 0.44)	0.41(0.04 to 0.77)^a^	-1.27(-4.56 to 2.02)	-1.76(-5.05 to 1.53)	0.004
BMI (kg/m^2^)	-0.82(-1.11 to -0.53)^a^	-0.86(-1.15 to -0.57)^a^	0.03(-0.11 to 0.18)	0.16(0.02 to 0.32)	-0.25(-1.42 to 0.91)	-0.45(-1.62 to 0.71)	0.002
WC (cm)	-1.68(-2.32 to -1.03)^a^	-1.98(-2.66 to -1.31)^a^	0.08(-0.52 to 0.35)	0.14(-0.31 to 0.59)	-1.99(-4.29 to 0.30)	-2.44(-4.75 to -0.13)^b^	0.016
TC (mmol/l)*	-0.08(-0.23 to 0.06)	-0.15(-0.32 to 0.01)	-0.16(-0.29 to -0.02)^a^	-0.15(-0.27 to -0.02)^a^	-0.02(-0.21 to 0.17)	-0.09(-0.27 to 0.09)	0.001
TG (mmol/l)*	-0.19(-0.34 to -0.04)	-0.24(-0.40 to -0.07)	-0.09(-0.21 to 0.02)	-0.03(-0.23 to 0.17)	-0.03(-0.21 to 0.14)	-0.09(-0.27 to 0.09)	0.003
HDL (mmol/l)*	0.10(0.06 to 0.14)^a^	0.12(0.08 to 0.15)^a^	-0.01(-0.03 to 0.02)	-0.12(-0.04 to 0.01)	0.09(0.02 to 0.16)^b^	0.12(0.05 to 0.19)^b^	0.043
LDL (mmol/l)*	-0.06(-0.22 to 0.09)	-0.16(-0.29 to -0.04)^a^	-0.07(-0.21 to 0.07)	-0.06(-0.19 to 0.07)	-0.12(-0.31 to 0.06)	-0.16(-0.35 to 0.03)	0.010
SBP (mmHg)*	-2.58(-4.89 to -0.27)^a^	-4.42(-6.76 to -2.07)^a^	1.31(-3.11 to 0.49)	-0.84(-2.36 to 0.67)	-0.73(-3.10 to 1.64)	-1.71(-3.97 to 0.56)	0.008
DBP (mmHg)*	-1.78(-3.16 to -0.39)^a^	-1.99(-3.22 to -0.76)^a^	0.59(-0.40 to 1.58)	-0.07(-1.12 to 0.97)	-2.39(-3.62 to -1.16)^b^	-2.63(-3.79 to -1.48)^b^	0.070
PA(MET-min/wk)	61.1(30.20 to 91.90)^a^	129.20(44.20 to 214.10)^a^	-5.40(-27.80 to 16.80)	-38.51(-78.8 to 1.80)	66.5(30.1 to 92.7)^b^	183.2(80.5 to 185.9)^b^	0.044
Energy (Kcal)	-32.5(-87.70 to 22.50)	-33.50(-86.30 to 19.30)	-23.10(-78.90 to 32.70)	27.10(-2.80 to 57.00)	-23.70(-87.5 to 40.15)	-34.60(-97.6 to 28.4)	0.004

Values are in mean (SD). Means differences within and between group are in mean (95% Confidence Interval), a negative change indicates a fall on average from baseline to 6 months and baseline to 12 months. Determined using repeated measures ANOVA

^a^ within groups comparison of mean difference, significant at p<0.05

^b^ between groups comparison of mean difference, significant at p<0.05

*adjusted for baseline value, co-morbidities and medication used.

#### Effects of Co-HELP on other cardio metabolic risk factors

There were significant differences between groups over time for diastolic blood pressure (mean difference, 95% CI) of -2.63 mmHg (-3.79 to -1.48) and HDL cholesterol level of 0.12 mmol/l (0.05 to 0.18), (p<0.01 for both) (Table 4). Further analyses demonstrated that the Co-HELP group had a decrease in diastolic BP by -1.78 mmHg and -1.99 mmHg at 6- and 12- month, respectively (p<0.001). In contrast, the usual care group showed an insignificant increase in diastolic BP by +0.59 mmHg and +0.07 mmHg at 6- and 12-month follow-up, respectively (p>0.05 for both). Relative to the usual care group, the intervention group showed significantly greater increase in the HDL cholesterol by +0.10 mmol/land +0.12 mmol/l (p<0.01) at 6- and 12-month follow-up. In contrast, the usual care group showed an insignificant decrease of HDL cholesterol by -0.01 mmol/land -0.02 mmol/lat 6- and 12-month, respectively (p>0.05 for both). Although there were no significant differences between groups over time for weight, BMI, systolic BP, total cholesterol, triglyceride or LDL cholesterol at 12-month, the pattern of change in some of these outcomes demonstrated meaningful positive results. Participants in the Co-HELP group showed positive changes in weight (-1.99 kg, -2.12 kg) and BMI (-0.82 kg/m^2^, -0.86 kg/m^2^) at 6- and 12-month, respectively (both p<0.001). Positive changes were also observed in the intervention group for waist circumference (-1.68 cm, -1.98 cm), total cholesterol (-0.08 mmol/l, -0.15 mmol/l), triglyceride (-0.19 mmol/l, -0.24 mmol/l), LDL cholesterol (-0.06 mmol/l, -0.16 mmol/l) and systolic BP (-2.58 mmHg, -4.42 mmHg) at 6- and 12-month respectively. On the contrary, the same parameters did not show meaningful positive changes in the usual care group.

#### Effects of Co-HELP on recommended targets of program

We further examined the percentage of participants who met recommended targets of program at 6-and 12-month follow-up (Table 5). At 6-month, 19.7% of the Co-HELP participants lost 5% or more of their initial weight compared to only 4.1% in the usual care group. The weight lost were maintained in the subsequent six months as 24.6% of participants in Co-HELP group met the recommended target compared to 3.4% in the usual care group(p<0.001). At 12-month, 9% of the Co-HELP participants progressed to normal BMI as compared to only 3.4% of participants from the usual care group. Also, at 12-month, 60.7% of Co-HELP participants were physically active (achieving of at least 600 METS-minute/week) as compared to 32.2% in the usual care group (p<0.001). Although statistical significance was not achieved in diet (p = 0.268), the Co-HELP group showed a greater percentage of participants (13.9%) who met the dietary aims (to reduce 20–25 kcal/kg energy intake) as compared to usual care group (9.6%).

**Table 5:** Percentage of participants who met clinical recommendation of lifestyle changes.

Clinical recommendations	Co-HELP(n = 122)	Usual care(n = 146)	p-value^a^
Weight from baseline			
Lost weight	96(78.7)	74(50.7)	<0.001
Stayed the same weight	3(2.5)	4(2.7)	
Gained weight	23(18.9)	68(46.6)	
Loss weight of ≥ 5%			
6-month	24 (19.7)	6 (4.1)	<0.001
12-month	30 (24.6)	5 (3.4)	<0.001
BMI classification			
6-month			
Normal (18.5 to 22.9 kg/m^2^)	12(9.8)	7(4.8)	0.093
Overweight (23.0 to 27.4 kg/m^2^)	38(31.1)	36(24.7)	
Obese (≥27.5 kg/m^2^)	72(59.0)	103(70.5)	
12-month			
Normal (18.5 to 22.9 kg/m^2^)	11(9.0)	5(3.4)	0.040
Overweight (23.0 to 27.4 kg/m^2^)	39(32.0)	36(24.7)	
Obese (≥27.5 kg/m^2^)	72(59.0)	105(71.9)	
Physical Activity			
6-month	49(40.2)	46(31.5)	0.140
12-month	74(60.7)	47(32.2)	<0.001
Dietary intake			
6-month	16(13.1)	16(10.9)	0.587
12-month	17(13.9)	14(9.6)	0.268

Values are n(%).

^a^ Determined by chi-square test comparison between two groups, significant at p<0.05.

#### Effects of Co-HELP on health-related quality of life

The HRQOL measures of the Co-HELP and usual care groups are presented in Table 6. At baseline, the mean scores of the SF-36 components were in the range of 60 to 90. At 12-month, the Co-HELP participants achieved improvement (mean difference, 95% CI) in the physical functioning (PF) with 14.41(10.32 to 18.49), role physical (RP) with 5.56(2.31 to 8.99), bodily pain (BP) with 10.64(5.77 to 15.52), general health (GH) with 15.69(12.52 to 18.85),vitality (VT) with 17.57(14.36 to 20.77), social functioning (SF) with 24.61(19.32 to 29.89), role emotion (RE) with 8.46(5.29 to 11.63), and mental health (MH) with 9.15(6.35 to 11.96), with all p values <0.001 as compared to the usual care group. Relative to the usual care group, participants in the Co-HELP group also showed significantly greater improvement in the Physical Component Score (PCS) with 6.51(5.21 to 7.80), (p<0,001) and Mental Component Score (MCS) 7.79(6.44 to 9.14),(p<0.001).

**Table 6:** Change in the health-related quality of life from baseline to12-month follow-up between groups.

SF36	Mean change (95% CI) from baseline to 12-month		Mean difference (95% CI) between groups	partial η^2^
	Co-HELP (n = 122)	Usual care (n = 146)		
PF	9.93(6.59 to 13.26)^a^	-6.74(-10.84 to -2.65)^a^	14.41(10.32 to 18.49)^b^	0.154
RP	9.69(6.01 to 13.37)^a^	1.21(-2.69 to 5.12)	5.56(2.31 to 8.99)^b^	0.040
BP	7.82(4.54 to 11.11)^a^	-3.57(-8.64 to 1.49)	10.64(5.77 to 15.52)^b^	0.065
GH	18.25(15.54 to 20.97)^a^	5.56(1.96 to 9.17)^a^	15.69(12.52 to 18.85)^b^	0.265
VT	4.42(1.79 to 7.06)^a^	-12.38(-16.02 to -8.74)^a^	17.57(14.36 to 20.77)^b^	0.305
SF	4.89(2.24 to 7.55)^a^	-20.97(-26.43 to -15.52)^a^	24.61(19.32 to 29.89)^b^	0.241
RE	6.73(3.42 to 10.04)^a^	0.11(-3.67 to 3.91)	8.46(5.29 to 11.63)^b^	0.095
MH	-2.13(-4.42 to 0.15)	-9.07(-12.14 to -5.99)^a^	9.15(6.35 to 11.96)^b^	0.135
PCS	4.29(3.13 to 5.45)^a^	-2.35(-3.87 to -0.84)^a^	6.51(5.21 to 7.80)^b^	0.271
MCS	2.22(1.26 to 3.18)^a^	-5.13(-6.67 to -3.59)^a^	7.79(6.44 to 9.14)^b^	0.328

Determined using ANCOVA.

^a^ within groups comparison of mean change, significant at p<0.05

^b^ between groups comparison of mean difference, significant at p<0.05. SF-36: PF: physical functioning, RP: role limitations due to physical health, BP: bodily pain, GH: general health, VT: vitality, SF: social functioning, RE: role emotions due to mental health, MH: mental health, PCS: Physical Component Summary and MCS: Mental Component Summary.

## Discussion

The purpose of this study was to determine the effects of the community-based healthy lifestyle intervention on diabetes risks and health-related quality of life among adults with prediabetes in the 6-month and 12-month follow-up. Our results indicated that the significant reductions in fasting blood glucose, 2-hour post glucose, HbA1C, waist circumference, diastolic blood pressure and an increase in HDL-cholesterol achieved during the first 6 months of the program were maintained at the 12-month as compared to the usual care group. Additionally, the HRQOL of the Co-HELP participants improved in both the physical and mental health components at 12-month. Thus, this study provides evidence that a community-based lifestyle modification intervention designed for prediabetes adults administered in collaboration with local community volunteers and NGOs can have significant and positive effects on risk factors of type 2 diabetes.

Although it is difficult to compare across studies with different baseline levels, it appears that the reduction in fasting plasma glucose in our study (-0.40 mmol/l) was comparable to the DPP study [11] (-4.0 mg/dl or -0.22 mmol/l) and FDPS study [13] (-5.0 mg/dl or -0.28 mmol/l), although they were conducted over longer follow up periods (2.8 years for DPP and 3.2 years for FDPS).

Not many translational studies of diabetes prevention in the community reported significant changes in blood glucose. For example, a study by Katula et.al [15] demonstrated that a community-based translational study of DPP among overweight and obese participants (BMI 25–40 kg/m^2^) with impaired fasting glucose (n = 301) showed significant decrease in FPG (-4.3 mg/dl or -0.25 mmol/l) after 12 months. Likewise, Oldroyd et.al [56], assessed the impact of lifestyle interventions in patients with impaired glucose tolerance (n = 78) reported a decrease in the 2-hour plasma glucose (-0.63 mmol/l) in intervention participants at 12 -month follow up. The study also reported that more participants in the intervention group reverted to normal glucose tolerance at 12–month than the control group; however, these differences were not statistically significant. In the present study, we also found similar results,22.1% in Co-HELP vs 4.1% in usual care participants reverted to normal glucose tolerance at 12-month (p>0.05). To our knowledge, this is the first diabetes translational study to document significant reductions in glucose metabolism measures in Malaysia.

The decrease in waist circumference in the Co-HELP group was in concordance with the results from other diet and exercise intervention studies [12, 57–59]. Decrease in the waist circumference indicate changes in body composition particularly with regards to the increase in muscle mass, decrease in fat mass, and improved glucose metabolism[60]. This finding is important to Asian populations who are known to be “metabolically obese” that poses a higher risk for type 2 diabetes [61]. The reduction in waist circumference could be due to increase in physical activity from low to moderate level in the Co-HELP group that may have induced the decreased in triglyceride (-0.24 vs. -0.03 mmol/l) and increased in HDL (0.12 vs. -0.02 mmol/l), as there were dose-response relationships between level of physical activity, HDL-cholesterol, and triglycerides [12, 60]. Although statistical significance between groups were not achieved, the mean weight loss in the Co-HELP group (-2.12 kg) was similar to the mean weight loss demonstrated in the systematic review and meta-analysis by Dunkley et al.[20] of -2.32 kg. This may be due to the fact that more participants in the Co-HELP group adhered to the program guidelines, particularly in achieving 5% or more weight loss and being physically active. A cross sectional study by Seppala et al.[29] in the community (n = 1383) demonstrated that IFG or IGT did not influence the participants’ health-related quality of life, but in a larger population study (n = 11,247) by Tapp et al. [62] reported that both IFG and IGT as well as newly diagnosed diabetes (NDM) were associated with reduced health-related quality of life. Thus, our results support the notions that community-based lifestyle intervention which incorporated physical activity, diet and behaviour modification strategies, can improve the physical and mental health component of the health-related quality of life outcomes among prediabetes individuals.

### Strength and limitations of the study

This study has a number of strengths. It provides evidence that a culturally adapted diabetes prevention program (DPP) can be implemented in the community setting, with reduction of several diabetes risk factors and improvement of health-related quality of life. Despite the pragmatic approach of intervention, our one-year results are encouraging. We were able to engage the communities and the group approach maximised efficiency. The collaborative effort and partnership among the participants, research team, community volunteers, local residential committees and healthcare providers enabled a sustainable approach to lifestyle modification change. Furthermore, the response rate of follow up measurements and data collection was relatively high (>85%) at 6-and 12-month.

In conducting community-based intervention, there are various limitations that need to be addressed. Clinical trials typically enrol highly motivated individuals that are probably more conscious of their own health condition than the general population. Such bias is often present and would be difficult to eliminate in majority of the studies evaluating lifestyle intervention. While a quasi experimental study design may be more feasible to conduct in the community setting, it may not sufficiently control the possibility of confounding factors due to lack of randomization. For example, although we made sure that the control group was comparable to the intervention group on the socio-demographics and blood glucose characteristics, it was not fully equivalent in terms of pre-existing hypertension and medication used. In the analysis, these baseline differences were taken into account and the comparisons between groups for blood pressure and lipid measurements should be interpreted with caution. We sought an alternative way to assess the impact of the lifestyle intervention on these outcomes by tracking changes in the usage of medications during their follow up. At 12-month, more than 10.6% of participants in the Co-HELP group were able to reduce their medication dosage as compared to only 3.8% of usual care group, indicating that the lifestyle intervention may have benefited them.

It is acknowledged that the use of carried forward data in the intention-to-treat analysis may result in an under or overestimation of the true outcomes. To address this problem, sensitivity analysis using complete cases were carried out. The results of this analysis were consistent with our earlier results, suggesting that the loss to follow up was not a major source of bias. As mentioned earlier, the treatment effects on most of the outcome measurements including HRQOL were comparable with the intention-to-treat analysis. We are also aware that there were lack of objective measurements on physical activity and dietary intake. However, self-reported questionnaires on physical activity and diet have also been used in other intervention trials [56, 63, 64] and were found to be reliable. The distance between both communities was approximately seven kilometres and the possibility that contamination occurred between the intervention and control groups cannot be ruled out. In order to limit “contamination” between these two groups, participants were followed up at their respective area or clinic on different days. Another limitation is that this study included participants from two sub-urban communities from a mid-size city in Malaysia. It is unknown whether this type of intervention can be implemented effectively in varying urban or rural settings involving various racial distributions. Future studies are recommended to determine whether similar outcomes could be achieved in communities with different socioeconomic background and geographical areas. It should also determine if the quality of life improvements can be sustained after the intervention is terminated.

## Conclusions

We conclude that a culturally adapted diabetes prevention program can be implemented in the community setting, with improvement in several diabetes risk factors and health-related quality of life. Collaborations with existing community partners demonstrated a promising channel for wide-scale dissemination of diabetes prevention at the community level.

## Supporting Information

S1 TREND checklist TREND checklist. (PDF)

S1 Fig Design and pathway of study phases. Design and study phases.
(TIF)

S1 Study protocol Study protocol for Co-HELP. (PDF)

S1 Table Sensitivity analysis using completes cases for outcome measures in the intervention and usual care groups. Sensitivity analysis of outcomes.
(TIF)

S2 Table Sensitivity analysis using complete cases for HRQOL in the intervention and usual care groups. Sensitivity analysis of HRQOL.
(TIF)
