# Supplementary figures and images for: Effects of a Community-Based Healthy Lifestyle Intervention Program (Co-HELP) among Adults with Prediabetes in a Developing Country: A Quasi-Experimental Study
Source: PLoS One. 2016 Dec 9;11(12):e0167123. doi: 10.1371/journal.pone.0167123 (PMC5147835; doi:10.1371/journal.pone.0167123)

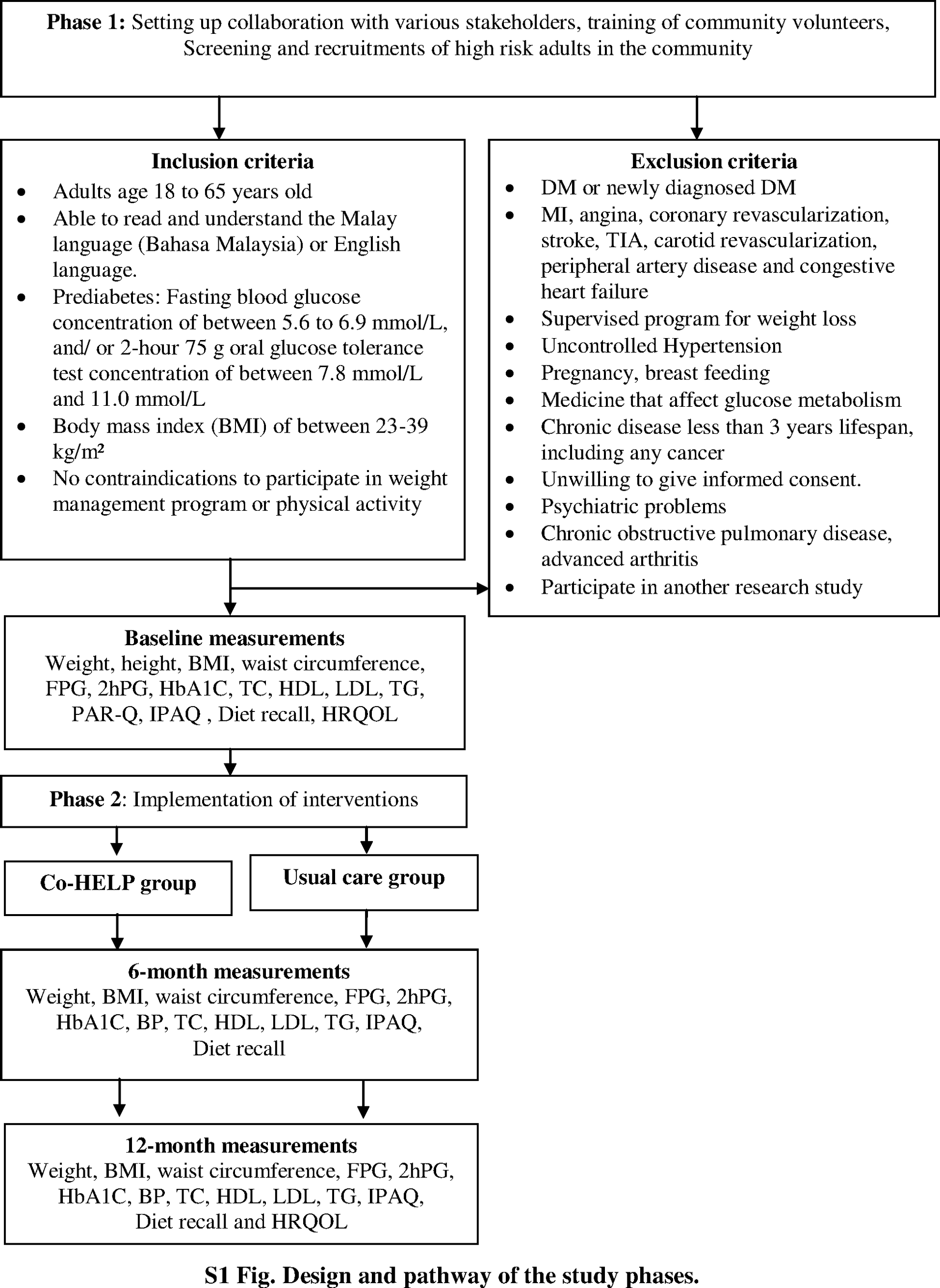

Supplement: S1 Fig — Design and study phases. (TIF) [file pone.0167123.s002.tif]

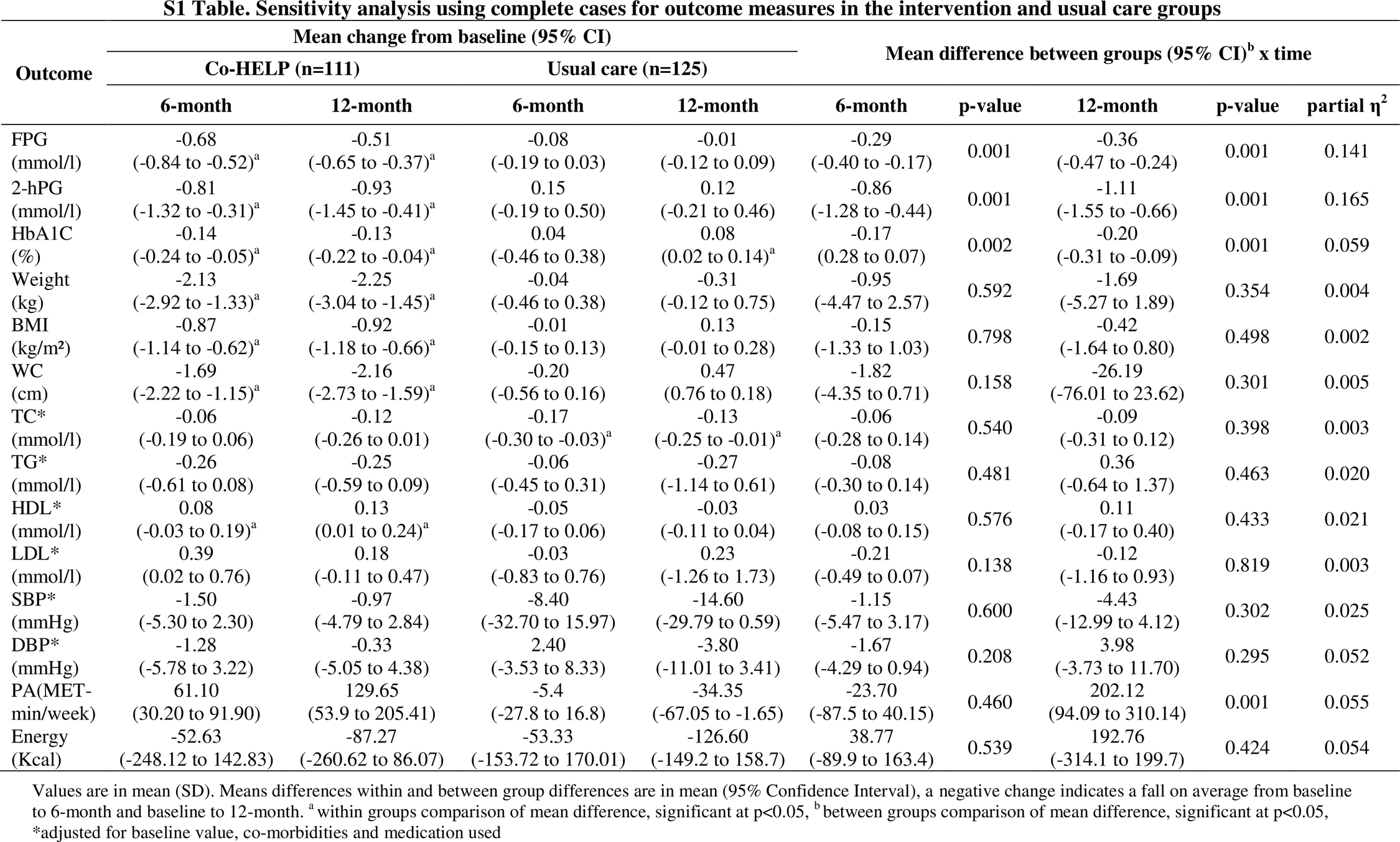

Supplement: S1 Table — Sensitivity analysis of outcomes. (TIF) [file pone.0167123.s004.tif]

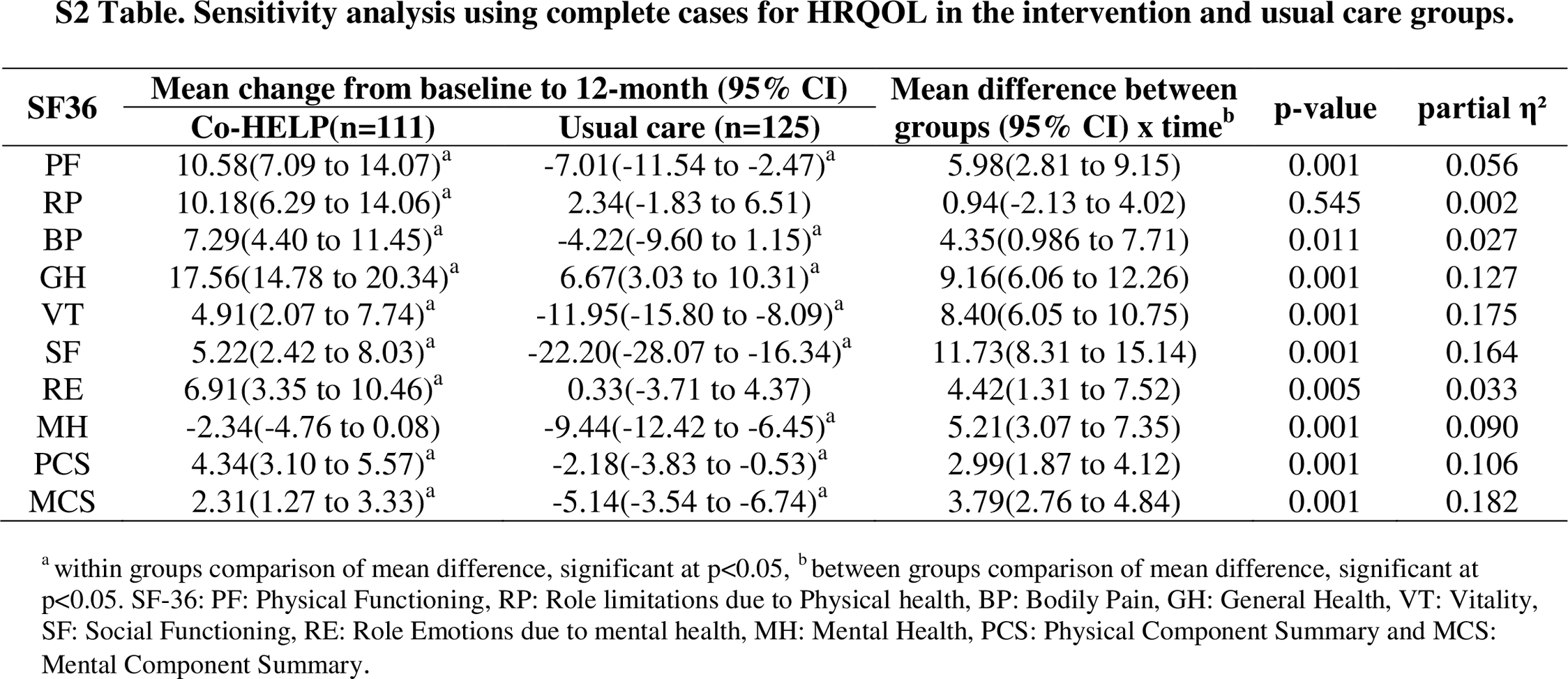

Supplement: S2 Table — Sensitivity analysis of HRQOL. (TIF) [file pone.0167123.s005.tif]
