# Supplementary material for: Effects of a Community-Based Healthy Lifestyle Intervention Program (Co-HELP) among Adults with Prediabetes in a Developing Country: A Quasi-Experimental Study
Source: PLoS One. 2016 Dec 9;11(12):e0167123. doi: 10.1371/journal.pone.0167123 (PMC5147835; doi:10.1371/journal.pone.0167123)
Supplement: S1 Study protocol — (PDF) [file pone.0167123.s003.pdf]

Research Protocol

"A community-based healthy lifestyle intervention amongst high risk individuals to prevent diabetes (Co-HELP)- a translational research."

By

DR NORLIZA BINTI IBRAHIM

DEPARTMENT OF SOCIAL AND PREVENTIVE MEDICINE

FACULTY OF MEDICNE, UNIVERSITY OF MALAYA

March, 2011

Table of contents:

1.0 Abstract

2.0 Introduction

3.0 Literature review

4.0 Study rationale

5.0 Objectives

- 5.1 General objectives
- 5.2 Specific objectives

6.0 Methodology

- 6.1 Study design
- 6.2 Study area
- 6.3 Study population
- 6.4 Conceptual framework
- 6.5 Sampling procedure
- 6.6 Sample size estimation
- 6.7 Flow chart
- 6.8 Case definition
- 6.9 Inclusion criteria
- 6.10 Exclusion criteria

7.0 Description of study interventions

8.0 How to ensure good response rate?

9.0 Study variables

10.0 Study measurements and instruments

11.0 Plan for data collection

12.0 Plan for data analysis

13.0 Study limitations and measures to minimize it

14.0 Ethical consideration

15.0 Public health impacts

16.0 Plan of work-‘Gantt chart’

17.0 References

Title : "Community-based healthy lifestyle intervention amongst high risk individuals to prevent diabetes (Co-HELP)- a translational research."

ABSTRACT

Background: Although recent evidence shown that lifestyle interventions are effective and can be delivered in an affordable manner in the community, more studies need to be done. This is due to the fact that more than 70% of people with diabetes live in low and middle income countries, and the largest increases in diabetes prevalence is predicted to occur in the Asian region. However, studies from developing countries are scarcely documented. Therefore, the objective of this study is to contribute to the growing body of literature on the effects of community-based lifestyle intervention among adults at high risk of type 2 diabetes.

Methods: This is a quasi-experimental study comparing a community-based healthy lifestyle intervention program (Co-HELP) to the usual care (UC) in high risk adults from two semi-urban communities. The population of this study is the high risk adults for type 2 Diabetes that will be recruited through the health screening in the community. The strategy of intervention is by using group-based approach, collaborative efforts with professionals in the initial stage but later on will be handled completely by community health workers and the peer educators. This is an innovative expansion of diabetes prevention program in the community setting that will determine the feasibility of Malaysia Diabetes Association as the empowered community partners.

Outcome measures: The primary outcome measures are change in the fasting blood glucose, 2-hour plasma glucose and HbA1C. Secondary outcome measures include weight, BMI, waist circumference, total cholesterol, triglyceride, LDL cholesterol, HDL cholesterol, systolic and

diastolic blood pressure, physical activity, diet and health-related quality of life (HRQOL). The outcomes will be analyzed at regular intervals within a period of one year.

Conclusion:

The finding from this study will perhaps demonstrate that a community-based lifestyle intervention administered through a local Non-Governmental Organization such as Malaysia Diabetes Association may become a promising strategy for widespread dissemination of interventions at preventing type 2 diabetes in high risk individuals.

1.0 BACKGROUND

Despite significant advances in Medicine, Diabetes Mellitus remains a major global health problem[1]. The number of people worldwide with diabetes is projected to rise from 285 million in year 2010 to 439 million in 2030. Between 2010 and 2030, there will be an increase of 70% of adults with diabetes in developing countries and 20% increase in developed countries[2]. The increasing prevalence of type 2 diabetes worldwide is due to combination of factors such as population growth, increasing life span, urbanization, increasing prevalence of obesity and physical inactivity. Malaysia is similarly affected due to a major shift in the lifestyles and longevity of the population[3]. This rising trend is mirrored in Malaysia where the prevalence of diabetes amongst adults became almost doubled between 1986 and 2006. Globally, this places Malaysia at number ten amongst countries with high diabetes prevalence [2]. The diabetes prevalence rate has risen much faster than expected as shown by National Health and Morbidity Survey (NHMS); 6.3% (NHMS I-1986), 8.3% (NHMS II-1996) and later was found to be 14.9% (NHMS III-2006). Based on this prevalence, the projected number of people age 18 years old and above affected by diabetes in Malaysia is estimated at 1.49 million. One in seven adults in Malaysia is a diabetic. What is even more worrying is the fact that almost half of our population with diabetes is unaware that they have the disease[4]. Evidence also suggests that diabetes mortality and diabetes-related complications continue to increase resulting in rising disease burden in the country [5]. These realities exist despite the success of large scale health campaign to enhance diabetes awareness in the community. Mere public forums and mass-media advertisements of healthy lifestyle have not been able to stem the rising tide of diabetes as shown in the past two decades[3].

Evidence from large scale clinical trials such as the United States Diabetes Prevention Program (DPP)[6] and the Finnish Diabetes Prevention Study (DPS) [7] studies have demonstrated

that lifestyle modification intervention is effective in reducing or delaying the onset of type 2 diabetes in high risk individuals by 40% to 58%. Although compelling evidence exists, it has yet to be widely adopted at the community level. Therefore, the current challenge is for us to translate these evidences that suit our local context and able to disseminate in long term. Collaborative efforts with the existing Governmental Organizations and Non-governmental organizations (NGOs) locally may help to fill the gaps and support the sustainability of the diabetes prevention program in the country. Due to our limited resources, emphasis is given on prevention of diabetes amongst first degree relatives of diabetic patients whom are pre-diabetes. The first degree relatives become an important target group because they can influence the lifestyle of other family members. Furthermore, the community needs to act more aggressively to reduce the modifiable risk factors of diabetes by healthy lifestyle intervention.

2.0 LITERATURE REVIEW

2.1 Epidemiology of Type 2 Diabetes Mellitus globally and different regions

Diabetes and associated complications pose a major health-care burden worldwide. It also presents the major challenges to public health and national economies[8]. The number of people developing Type 2 diabetes is rising dramatically worldwide. This is evident from findings of a series of global estimates of current and predicted future prevalence of diabetes [9-12] as shown in table 1.

Table 1: Estimates of current and projected global prevalence of diabetes in adults

	**King et al. (1998)**		**Int. Diabetes Fed.(IDF) (2003)**		**Wild et al. (2004)**		**Shaw et al. (2009)**	
**Adults**	Current (1995)	Projected (2025)	Current (2003)	Projected (2025)	Current (2000)	Projected (2030)	Current (2010)	Projected (2030)
**Prevalence**	4%	5.4%	5.1%	6.3%	2.8%	4.4%	6.4%	7.7%

Numbers	135 million	300 Million	194 million	334 million	171 million	366 million	285 million	439 Million

Based on current trends, it was predicted that the population of diabetes will swell up to a staggering 439 million by the year 2030, of which almost half of that will be from the South Asia and Western Pacific Region. Every region will have an increase in numbers as well as excess of adult population growth, thus total number of diabetes are likely to increase by 50% over the 20 years [12]. The varying prevalence rates for Type 2 DM were observed in different parts of the world which reflects the underlying differences in ethnicity, demography and social risk factors such as lifestyle, diet, obesity prevalence and physical inactivity [13].

About 80% of people with diabetes are in developing countries, with Asia being the major site of rapidly emerging diabetic epidemic[11]. One of the hallmarks of diabetes in Asian countries is the rapidly increasing prevalence of young-onset diabetes (people younger than 44 years old)[8]. Factors that have contributed to the epidemic of obesity and young-onset diabetes are the rapid transition in dietary habits, reduced physical activity, changing pattern in leisure activities, longer working hours and decreasing sleep hours[14]. Type 2 diabetes has a strong genetic component and most Asian patients have first-degree relative with diabetes[15]. Prevalence estimates (standardized to world age) of diabetes and impaired glucose tolerance in all Asian countries are high and are expected to increase further during the next two decades[8].

2.2 Epidemiology of Type 2 Diabetes Mellitus in Malaysia

Malaysia is similarly affected due to a major shift in the lifestyles and longevity of the population[3]. This rising trend is mirrored in Malaysia where the prevalence of diabetes amongst adults became almost doubled between 1986 and 2006. The diabetes prevalence rate has risen much faster than expected as shown by National Health and Morbidity Survey (NHMS); 6.3% (NHMS I-

1986), 8.3% (NHMS II-1996) and later was found to be 14.9% (NHMS III-2006). Based on this prevalence, the projected number of people age 18 years old and above affected by diabetes in Malaysia is estimated at 1.49 million. One in seven adults in Malaysia was a diabetic. What is even more worrying is the fact that almost half of our population with diabetes is unaware that they have the disease[4]. The status of Diabetes Mellitus in Malaysia for the past two decades is shown in Table 2.

Table 2 : Status of Diabetes Mellitus in Malaysia (1986-2006)

**Parameters**	**NHMS I (1986)**	**NHMS II (1996)**	**NHMS III (2006)**	**NHMS III (2006)**
Age group (years)	≥ 35 years	≥ 30 years	≥ 18 years	≥ 30 years
Prevalence of diabetes (overall)	6.3%	8.3%	11.6%	14.9%
Prevalence of known diabetes	4.5%	6.5%	7.0%	9.5%
Prevalence of newly diagnosed diabetes	1.8%	1.8%	4.5%	5.4%
Prevalence of pre-diabetes<br>IGT*<br>IFG**	4.8%*	4.3*	4.2**	4.7**

*based on IGT , **based on IFG

Several factors may account for the higher prevalence of diabetes in Malaysia. Although the molecular basis for type 2 diabetes is still poorly understood, but both insulin resistance and beta-cell dysfunction are well documented [16] and is likely as a result of both environmental influences and genetic factors [17]. Food rich in carbohydrates and saturated fat is a characteristic feature of Malaysian diet. Coupled with modernization and physical inactivity have impacted our population which appears to be genetically predisposed to Type 2 Diabetes[3].

2.3 Pre-diabetes or borderline diabetes

Type 2 Diabetes Mellitus is a complex metabolic disorder due to insulin resistance and or defects in insulin secretion. At the time of diagnosis, only about 50% of β cell function remains which underscore the progressiveness of the disease and importance of early detection and prevention[18]. Type 2 diabetes usually preceded by a condition known as 'pre-diabetes' which refers to a metabolic stage that is intermediate between normal glucose homeostasis and diabetes - impaired glucose metabolism. These condition referred to patients who have either impaired glucose tolerance (IGT) or impaired fasting glucose (IFG) in which individuals have blood glucose levels that are higher than normal but not high enough to be classified as diabetes [19]. Impaired fasting glucose (IFG) is identified by fasting plasma glucose (no caloric intake for ≥ 8 hours) measurement between 5.6-6.9 mmol/L (100-125 mg/dL), whereas impaired glucose tolerance (IGT) is identified by an oral glucose tolerance test (OGTT) in which the 2 hour post prandial (75 gm glucose load) measurement is between 7.8-11.0 mmol/L (140-199 mg/dL). People with pre-diabetes are at increased risk of developing diabetes, cardiovascular diseases and other obesity-related adverse health outcomes[20]. Both Impaired Glucose Tolerance (IGT) and Impaired Fasting Glucose (IFG) are known to predict subsequent diabetes. Between 33% and 65% of those with IFG and IGT may go on to develop type 2 diabetes within 6 years, compared to less than 5% of those with normal blood glucose level[21]. In other studies, people with IGT have been shown to have risk developing diabetes of 4.3% per year among Chinese ethnic in Singapore[22], 8.8% per year in Taiwan [23] and 11.2% in China [24]. The third National Health and Morbidity Survey (NHMS III) conducted in 2006, showed that the national prevalence of IFG amongst Malaysian adults was found to be 4.2% (CI: 4.0-4.5), with highest prevalence in the states of Wilayah Persekutuan Kuala Lumpur (6.1% (CI: 4.8-7.5)), Pulau Pinang (6.1% (CI : 5.0-7.1)) and Perak (5.0% (CI: 4.0-6.0)). The prevalence of IFG

was significantly higher among the urbanities (4.5% (CI: 4.2-4.9)) than rural (3.8% (CI: 3.4-4.1)) and in males (5.2% (CI: 4.8-5.6)) as compared to females (3.5% (CI: 3.2-3.8)) [5].

2.4 Studies of lifestyle interventions to prevent type 2 diabetes

Over the past decade, several Randomized Control Trials have formally tested the hypothesis that lifestyle modification can prevent type 2 diabetes.

i) Chinese Da Qing IGT and Diabetes Study

The first of these studies was conducted in Chinese community health clinics and included 577 individuals with impaired glucose tolerance called as the Da Qing IGT and Diabetes Study [24]. Individuals were assigned according to which clinic they attended to a control group and three active treatment groups : diet only, exercise only or diet and exercise, They were followed up for 6 years. The dietary only intervention focused on increased consumption of vegetables and reduced consumption of alcohol and simple carbohydrates; overweight individuals with BMI ≥25 kg/m² were also advised to limit their calorie intake. Participants in the exercise only group were encouraged to increase their daily activity by equivalent of at least 20 min of brisk walking. The diet plus exercise group received both interventions and patients attended usual care clinics served as a control group. The 6-year cumulative incidence of diabetes was high in all groups (48%, 41%, 46% and 68% in the diet-only, exercise-only, diet plus exercise and control groups, respectively). The relationship between the amount of weight lost and diabetes incidence was inconsistent and all three interventions were similarly effective in preventing diabetes. The active intervention groups associated with 31%, 46% and 42% reduction in the risk of developing diabetes respectively.

Interestingly, the greatest reduction in the risk of developing diabetes occurred in the exercise group, underscoring the importance of physical activity in any diabetes prevention program.

ii) Finnish Diabetes Prevention Study (DPS)

The Finnish Diabetes Prevention Study (DPS) [7], randomized 522 over weight patients, middle-aged adults with IGT to intervention lifestyle group or control group. The lifestyle intervention included dietary and exercise components. The weight-loss goal was 5% or more of baseline weight. To achieve this target, the participants were instructed to reduce fat intake and increase consumption of fiber, whole grains, vegetables and low-fat dairy products. The exercise component required participants to do moderate-intensity exercise for at least 30 min per day. The intervention group lost an average of 4.2 kg in the first year of the study compared with the control group who had a 58% reduction in diabetes incidence during the whole study (~4 years). Although this study was not designed to assess the individual contributions of the diet and exercise components, participants who undertook particularly vigorous leisure-time physical activities had a markedly reduced risk of diabetes. After cessation of the intervention, the reduction in diabetes risk persisted—over 7 years of follow-up, the intervention group had an overall reduction in their risk of diabetes of 43% compared with the control group; during the post intervention period, the risk reduction was 36% in the subset of participants who were free from diabetes at the end of the intervention period despite modest weight regain (1.3 kg regained of the original 3.1 kg lost by this subgroup) in the intervention group. As expected, individuals who maintained the changes to their lifestyle during the extended follow up period had the greatest reduction in their risk of diabetes (a 46% risk reduction compared with those who did not achieve their goals). In a multivariate analysis

that considered the success of achieving all five lifestyle goals, only weight reduction remained a significant determinant of diabetes risk.

iii) American Diabetes Prevention Program (DPP)

The largest and most comprehensive lifestyle modification study was the US Diabetes Prevention Program (DPP) [6]. The DPP randomly allocated 3,234 overweight, mostly middle-aged adults with impaired glucose tolerance and fasting plasma glucose values of 5.3 mmol/l or higher to intensive lifestyle intervention, metformin or placebo. An additional 585 participants were randomly assigned to troglitazone therapy, but recruitment to this group and the participants' ongoing treatment were terminated prematurely owing to concern over possible hepatotoxic side effects. Troglitazone was not approved as a study drug in some locations that had enrolled American Indian participants. The DPP preferentially enrolled individuals at high risk of developing type 2 DM of which 45% of participants were from high-risk ethnic groups and 20% were aged 60–85 years at baseline. The DPP intensive lifestyle intervention was intended to achieve a 7% loss in body weight over 24 weeks. Participants were instructed to perform 150 min moderate-intensity physical activity (such as brisk walking) per week and to eat a low-fat, reduced-calorie diet. They also received one-to-one counseling to aid these changes in behavior that was continued (generally monthly) after the initial 24-week period to help maintain weight loss and activity levels. The lifestyle-intervention group achieved a mean weight loss of 7% (an average of 7.0 kg) within the first year, and had an overall mean weight loss of 5.6% (an average of 5.6 kg) during follow up (mean duration 2.8 years). The physical activity goals were met by 74% of participants in the first 24 weeks of the study. The DPP lifestyle intervention was associated with a 58% reduction in the incidence of diabetes, compared with placebo plus standard lifestyle recommendations. Weight loss was the predominant

predictor of reduced diabetes incidence, with a 16% reduction in risk per kilogram of weight lost. However, participants who achieved their exercise goals but not their weight loss goals also experienced some reduction in diabetes risk (44%) as compared with placebo-treated individuals. The effectiveness of the DPP lifestyle intervention was similar in all ethnic groups, both sexes and was greatest in older participants aged 60–85 years. Changes in physical activity, diet and weight loss were associated with reduced risks of developing type 2 DM.

iv) Diabetes prevention studies in Japan and India

Diabetes prevention studies in Japan [25] and India [26] also confirmed that the benefit of lifestyle modification interventions. Even with quite modest weight losses (0–2 kg), risk reductions of 67% and 28% were achieved in the Japanese and Indian studies, respectively. The differences in study design and participant characteristics make direct comparisons difficult, but these findings do seem to support the effectiveness of lifestyle modification across a broad range of ethnic groups and cultures.

2.5 Other translational research

The translational research transforms currently available knowledge into useful measures for everyday clinical and public health practices. It enhances adoption of the best practices in the community. Earlier, many published translational studies on diabetes prevention used quasi experimental designs for evaluation. Some community-based studies that preceded randomized clinical trials such as the DPP and DPS focused on implementation of school-based programs designed to increase physical activity and improve health awareness in young people. Results of these studies were varied, but suggested that these interventions improved participants’ knowledge

about healthy lifestyle choices and increased their adoption of risk-reducing behaviors, although effects on diabetes development have not been reported. Studies in adults have focused primarily on interventions that increase levels of physical activity and promote consumption of a healthy or a weight-loss diet. In general, improvements were seen in rates of physical activity and some groups experienced weight loss, but these changes generally did not reach significance because of the study designs used, including the lack of control groups. Finland has implemented a national program called FIND2D (Finnish type 2 Diabetes Prevention Plan 2003–2007). The program was being conducted in five districts' hospitals and covered a population of 1.5 million people. The most recent evidence showed that DPP lifestyle intervention can be translated into community settings is offered by the work of Ackermann et al, 2008 [20]. This study used the Young Men's Christian Association as a delivery site for implementation of the DPP intensive lifestyle intervention. The researchers studied a group based version of the DPP curriculum in a randomized clinical trial. They reported significant weight loss in the intervention group and was sustained at 12 months after the study has ended.

2.6 Meta-analysis on 9 randomized controlled trials on efficacy of lifestyle education to prevent type 2 diabetes

Lifestyle education was effective for reducing both 2-hour plasma glucose and Relative Risk (RR) in high risk individuals and may be useful tool in preventing diabetes. This was proven by a meta-analysis on 9 randomized controlled trials of efficacy of lifestyle education interventions for preventing type 2 diabetes in high risk individuals [27]. The results showed that lifestyle education intervention reduced 2 hour plasma glucose by 0.84 mmol/L (95% CI : 0.39-1.29) compared to the

control group. The one year incidence was reduced by 50% (RR 0.55, 95% CI; 0.44-0.69) compared with the control groups.

3.0 STUDY RATIONALE

i) Evidence from studies have demonstrated that lifestyle modification intervention is effective in reducing or delaying the onset of type 2 diabetes in high risk individuals by 40% to 58%[6]. Studies were done as Randomized Control Trials (RCTs), Cohort and Quasi Experimental studies with duration between 6 months to several years. However, the model of lifestyle interventions and the target groups (ie: high risk individuals or overt type 2 diabetes) varies from country to country. In Malaysia, although there were many studies done on diabetes but they were mainly prevalence studies that described the distribution and awareness of diabetes in the population [5, 28-31]. To date, no randomized controlled trials have been conducted to investigate the effectiveness of translating diabetes prevention program into the community. Moreover, it is not known whether a structured lifestyle modification intervention is superior to the usual care in promoting better health outcomes in Malaysia. Therefore, there is a need for a pragmatic randomized controlled trial to investigate which program of intervention yields the best outcomes for patients.

ii) The most recent evidence showed that lifestyle intervention can be translated into community settings by Ackermann et al, 2008 [20]. The Young Men's Christian Association was used as a delivery site for implementation of the intensive lifestyle intervention. They reported significant weight loss in the intervention group and was sustained at 12 months after the study has ended. Successfulness of translating the diabetes prevention program in non-health facility is a promising finding that can be tested in Malaysia. Therefore, by conducting this study we will determine the lifestyle modification interventions that suit our local context.

iii) Type 2 diabetes usually preceded by a condition known as ‘pre-diabetes’ which refers to blood glucose levels that are higher than normal but not high enough to be classified as diabetes [19]. People with pre-diabetes are at increased risk of developing diabetes, cardiovascular diseases and other obesity-related adverse health outcomes[20]. Between 33% and 65% of those with Impaired Fasting Glucose (IFG) and Impaired Glucose Tolerance (IGT) may go on to develop type 2 diabetes within 6 years, compared to less than 5% of those with normal blood glucose level[21]. The third National Health and Morbidity Survey (NHMS III) conducted in 2006, showed that the national prevalence of IFG amongst Malaysian adults was found to be 4.2% (CI: 4.0-4.5), with highest prevalence in the states of Wilayah Persekutuan Kuala Lumpur (6.1% (CI: 4.8-7.5)). The prevalence of IFG was significantly higher among the urbanities (4.5% (CI: 4.2-4.9)) than rural (3.8% (CI: 3.4-4.1)) and in males (5.2% (CI: 4.8-5.6)) as compared to females (3.5% (CI: 3.2-3.8)) [5]. Due to our limited resources, emphasis should be given on prevention of diabetes amongst high risk group by means of lifestyle modification intervention and screening for pre-diabetes. This has been strongly recommended by the International Diabetes Federation[32].

iv) Although health care professionals are making attempts to increase the public’s awareness about the benefits of lifestyle approach, however not many of them actually deliver it during their practice.

v) There are sufficient guidelines and policies available, however the main challenge we have to face is to translate them into real practice in our community.

vi) This is an innovative expansion of diabetes prevention education program in the community setting that will determine the feasibility of Malaysian Diabetes Association or any other local NGOs as the empowered community partners.

4.0 OBJECTIVES

a) General objective:

The general objective of this study is to assess the effectiveness of community-based lifestyle modification intervention as compared to usual care (conventional intervention) in reducing the modifiable risk factors of type 2 diabetes in high risk adults.

b) Specific objectives:

i) Primary objective-to determine the changes in blood glucose (FPG, 2hPG and HbA1C) among adults who received community-based lifestyle modification intervention as compared to the usual care intervention.

ii) Secondary objective-to determine the changes in other cardiometabolic risk factors (ie; total cholesterol, HDL cholesterol, Triglycerides, Blood Pressure, weight, BMI, waist circumference), dietary intake, physical activity and health-related quality of life (HRQOL) among adults who received community-based lifestyle modification intervention as compared to the usual care intervention..

5.0 METHODOLOGY

5.1 Study design

This is a Quasi-experimental study with repeated measures, collaboration between Department between the research team from Department of Social and Preventive Medicine, University of Malaya and community volunteers from Malaysian Diabetes Associations (MDA), local residential committees and primary health clinics.

5.2 Study area

The study will be conducted in two sub-urban communities in Seremban, Negeri Sembilan, Malaysia. Based on 2010 report [33-35], two communities i.e., Ampangan and Senawang with similar demographic and socioeconomic characteristics were selected.

5.3 Study population

The population of this study is the high risk adults for type 2 Diabetes. They will be recruited to participate in this study through screening in the community-setting.

5.4 Framework of the ‘high risk approach of diabetes prevention as below:

“The high risk approach” (as recommended by International Diabetes Federation [32]).

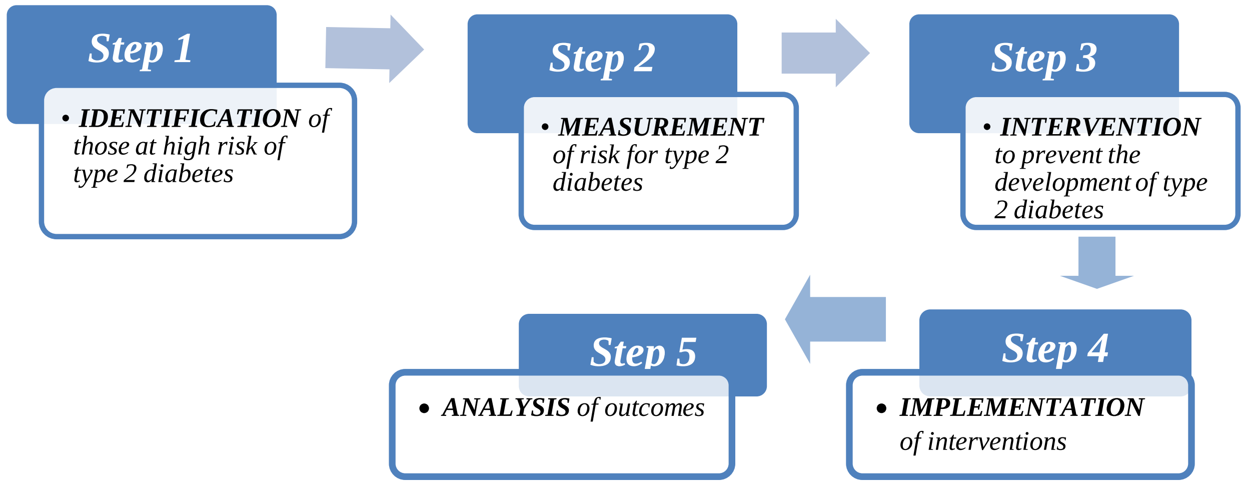

i) Step 1: Identification of those who may be at higher risk of type 2 diabetes by using simple, practical, non invasive and inexpensive methods.

The IDF recommend the use of brief questionnaires to help health-care professionals to quickly identify people who may be at higher risk and who need their level of risk further investigated. This type of questionnaire could also be used by individuals for self-assessment. This simple diabetes risk score is based on a set of variable not requiring laboratory tests, can be used as a practical tool to predict type 2 diabetes risk or to identify undetected type 2 diabetes. Examples are the Finnish Type 2 Diabetes Risk Assessment Form [36] , American Diabetes Association Diabetes Risk Test [37], Thailand Modified Diabetes Risk Score[38] or Cambridge Scoring System [39]. However, there is still no validated or translated questionnaire for Malaysians. Hence, the screening of high risk individuals for this study will follow the screening procedures that are recommended by the Ministry of Health [40].

ii) Step 2: Measuring the risk for the development of type 2 diabetes.

If a person is considered to be at higher risk for development of type 2 diabetes, they will proceed to step 2 which is the measurement of risk by a health-care professional. The key investigation is the measurement of blood glucose. The presence of IGT or IFG gives a considerably increased risk of developing type 2 diabetes. Other risk factors for diabetes that should also be assessed at this stage include the presence of family history of diabetes, increased waist circumference, high blood pressure, raised triglycerides or pre-existing cardiovascular disease.

Table 3: Other high risk factors for type 2 diabetes

Family history of diabetes	First degree relative with diabetes
Waist circumference	≥ 90 cm for men and ≥ 80 cm for women
Overweight	BMI > 23 kg/m^2
Dyslipidemia	Either High Density Lipoprotein (HDL) cholesterol < 0.9 mmol/L or Triglycerides (TG) > 1.7 mmol/L
Hypertension	Blood pressure > 140/90 mm/Hg or on medication
Pre-existing cardiovascular diseases	Ischemic heart disease, cerebrovascular disease, peripheral arterial disease
Physically inactive	Sedentary work
High risk women	Women with history of polycystic ovarian syndrome (PCOS)

iii) Step 3: Intervention to lower the risk.

There is substantial evidence that lifestyle modification intervention can help prevent the future development of type 2 diabetes [6-7, 41]. Weight loss will improve insulin resistance, hyperglycemia, dyslipidemia and hypertension conditions in short-term. The aim is gradual weight loss (0.5-1.0 kg per week) through moderate calorie restriction (reducing calorie intake by 500-1000 calorie) and increased physical activity. A structured approach can produce long-term weight loss of 5-7% of baseline body weight[6]. Physical activity is important in maintaining weight loss, improves insulin sensitivity, improves dyslipidemia, general cardiovascular health and lowers blood pressure. Exercise programmes should start slowly and gradually build up. The IDF recommends a goal of at least 30 minutes of moderate physical activity (i.e., brisk walking, swimming, cycling, dancing) on most days of the week. When lifestyle intervention alone is not sufficient to achieve the desired weight loss or blood glucose, pharmacological interventions should be considered as a diabetes prevention strategy.

iv) Step 4: Implementation of interventions

Implementation of interventions follow the group based approach.

v) **Step 5: Analysis of outcomes**

The outcome measurements can be determined as short term or long term outcomes

5.5 Recruitment of high risk adults

Adults with high risk of developing type 2 diabetes will be invited to attend community-based screening and education program. The participants were recruited from general population through presentations at community-halls, mosques, as well as through media exposure like radio and Malaysian Diabetes Association website, brochures, banners, posters and healthcare providers. Screening of the high risk adults will be done in cooperation with Malaysian Diabetes Association staffs. All people attending the screening program will receive pamphlets regarding diabetes, risk factors, healthy food choice, exercise and etc. The screening tools that will be used are among the easy to perform, inexpensive, non invasive and able to sustain in the community (i.e.; Diabetes risk score questionnaire, capillary plasma glucose, weight and height for Body Mass Index (BMI), waist circumference and blood pressure).First they will have to complete the screening questionnaire. A drop of whole blood by finger prick will be done to assess Random Blood Glucose for people with BMI $\geq 23 kg/m^2$. Asymptomatic individuals with Random Blood Glucose $\geq$ 6.0 mmol/L will be inform that they are at risk of developing diabetes and are potentially eligible for the study. Participants will be given an appointment date for baseline investigation at different dates to avoid contamination.

During baseline investigation, the participants will be assessed for Fasting Venous Plasma Glucose (FPG) and or Oral Glucose Tolerance test (OGTT). The algorithm of the screening process and diagnostic values will be based on Clinical Practice Guidelines by Ministry of Health Malaysia on Management of Type 2 Diabetes Mellitus, 4th edition 2009[40]. People with fasting plasma

glucose between 5.6 to 6.9 mmol/L(100-125 mg/dL) or 2-hour plasma glucose between 7.8 to 11.0 mmol (140-199 mg/dL) will be informed that they are at increased risk of developing diabetes and are eligible to participate in the study. However, for people whose fasting plasma glucose $\geq$7.0 mmol/L or 2-hour plasma glucose $\geq$ 11.1 mmol/L will be refer to the health care provider immediately for follow up. Other measurements include waist circumference, weight, height, Body Mass Index (BMI), blood pressure, brief dietary assessment and physical activity assessment. These people will be enroll in the study if they agree to provide a written consent, free from any co morbidity that is contraindicated for light to moderate physical activity.

5.6 Allocation of participants

The study participants will be allocated to the intervention or control group depending on which community they were enrolled at the time of the baseline screening.

5.7 Analysis of outcomes

The participants will be assessed at baseline, 6th and 12th months after interventions given to them. The plan for data analysis will be described further in subsequent section.

5.8 Sample size estimation

Refering to the study by Kulzer et al [42] using a group based lifestyle intervention study to prevent diabetes mellitus; mean weight loss and duration of physical activity per week were significantly higher while fasting glucose was significantly lower in the intervention group compared to the control group after 12 months follow up. Using the sample size calculation from STATA

program, with power of 80% and significant level preset at 0.05, using different indicators, the calculated sample sizes are as the following:

	Duration of physical activity (minutes)	Fasting blood glucose (mg/l)	Weight loss (kg)
Mean difference 1	17.9	1.8	3.8
Mean difference 2	46.6	-4.3	1.4
Standard deviation 1	63.8	13.1	5.2
Standard deviation 2	95.5	11.3	4.1
Calculated Sample size group 1	122	64	60
Calculated Sample size group 2	122	64	60
Final sample size (with 30% attrition)	122 x 2 = 244	83 x 2 = 166	78 x 2 =156

Based on the above, the largest sample size will be used in this proposed study to ensure adequate power for most outcome measures. Therefore, 244 subjects will be require for this study.

5.9 Flow chart of sampling procedure

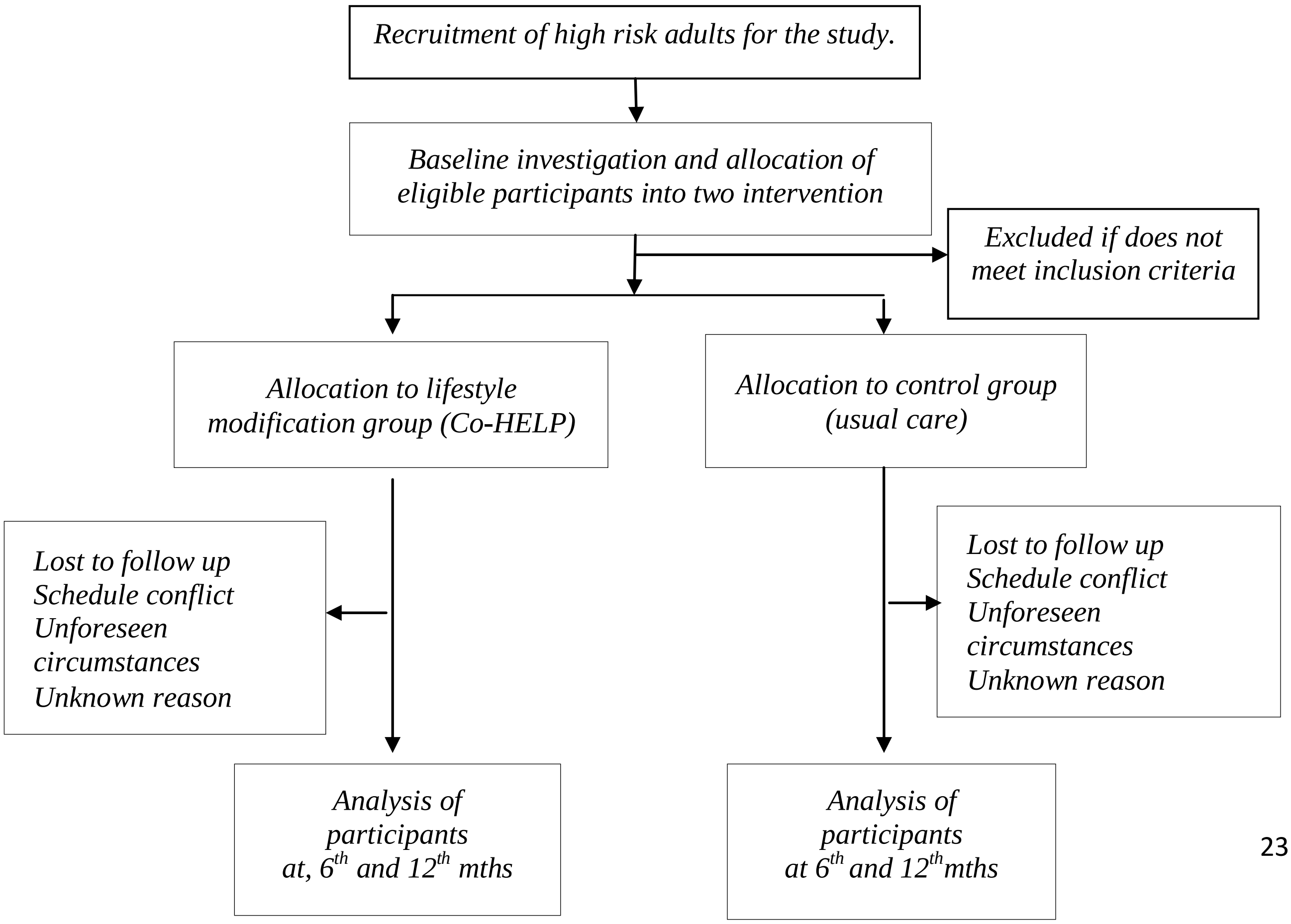

5.10 Case definition

i) Inclusion criteria

Participants are eligible if they live or work within the community area, age between 18- 65 years old, able to read and understand Bahasa Malaysia or English language, are at risk of type 2 diabetes with fasting blood glucose concentration of between 5.6 to 6.9 mmol/L, and/ or 2-hour 75 g oral glucose tolerance test concentration of between 7.8 mmol/L and 11.0 mmol/L [19], Body mass index (BMI) of between 23-39 kg/m² and had no contraindications to participate in weight management program or physical activity.

ii) Exclusion criteria

Had clinical history of diabetes or newly diagnosed diabetes at the time of screening, involved in other weight management program, took any herbal medications to reduce weight, had clinical history of cardiovascular diseases occurred within the past 6 months, advanced arthritis, uncontrolled hypertension, any form of cancers that require treatment, pregnant and other causes which could interfere with participation (i.e., psychological disorder or physically disabled).

6.0 DESCRIPTION OF STUDY INTERVENTIONS

6.1 Phase 1:

i) Setting up collaborations between partners

Collaboration with different study partners is very important to ensure the successfulness of this study. Therefore, the first important step is to advocate the study partners (i.e.; MDA, SPM and local residents) about the purpose and benefit of this study. This will be done in the form of formal

and informal meetings with the study partners with the aim to gain an agreement and support throughout this study period.

ii) Training of trainers (TOT)

After obtaining the agreement from the study partners, a one day training session for trainers will be conducted. The contents of the training session will include an update on type 2 diabetes information and importance of diabetes prevention at pre-diabetes stage, detail explanation of Co-HELP study, dietary modification and weight reduction strategy, physical activity and roles of community volunteers. This session will be conducted as lectures or in a more interactive way such as problem-solving skill and role-play. The trainers for this study are among health professionals..

6.2 Phase 2:

i) Implementation phase

This phase will include the various stages as mentioned earlier such as; i) recruitment of high risk adults, ii) screening of high risk adults for eligibility, iii) allocation of participants to either intervention group and usual care group and v) implementation of intervention to both the groups. The community-based healthy lifestyle intervention program (Co-HELP) was designed following review of current evidences [6-7, 24, 43-48] and guidelines [40, 49-50]. The group-based programs was adopted from publicly available materials from the US Group Lifestyle Balance of Diabetes Prevention Program (DPP Research Group) [51], combined with materials from Integrated Educational Module for Non-Communicable Diseases from Ministry of Health Malaysia[52]. Modification was tailored to the needs of the community and cultural sensitivity. Few consultations were done with stakeholders (i.e. dietitian, physical trainers, family medicine specialist, local

community organizations and potential participants) prior to interventions. The education materials were refined and pre-tested on the community-volunteers prior to delivery. The content focus was based on the Health Belief Model approach as its theoretical framework[53]. The 12-month intervention program consisted of two parts comprising of a 6-month active period and a 6-month maintenance period. In total, participants in the intervention group received twelve group-based sessions and minimum of two individual counseling with the dietitian to reinforce behavioural change. Most of the group sessions were held during weekends, at convenient community settings like community hall and recreational park. The first 6-month active period consisted of structured 90 minutes group sessions; biweekly in the first 3 months (Sessions 1-6) and monthly in the next 3 months (Sessions 7-9). During the maintenance period the group sessions were held monthly for three months (Session 10-12) and for the last three months participants were followed up through telephone calls or home visits. All intervention sessions were conducted either in groups of 20 people or smaller groups of 5 – 8 people depending on the type of activities (i.e. lecture, seminar, group work or discussion). Table 1 provides detailed information on session titles, frequency, content focus and the Health Belief Model constructs.

ii) Intervention group

The lifestyle modification intervention sessions will be conducted in group format, coordinate and facilitate by the community volunteers. The intervention will focuses on empowerment of participants to change their lifestyle. This intervention will incorporates diet, physical activity and healthy behaviour modification. The contact schedule occurs in three phases, with an early 3- month intensive phase (1 group session every 2 weeks) followed by a 6-month non-intensive phase (1 group session every month) and a 3-month maintenance phase. The goals set for

this intervention is based on what is outlined in the Clinical Practice Guidelines by Ministry of Health Malaysia, Management of type 2 Diabetes Mellitus, 4th edition 2009[40], to produce modest but yet achievable outcomes; reduction of 5-10% of initial body weight for all overweight and obese participants, reduction of calorie intake (20-25 kcal/kg body weight) and increase light to moderate physical activity to at least 150 minutes/ week. The group will have series of different sessions with professionals like Physicians, Dietician/ Nutritionist and Physical trainer specialist during the intensive phase. Throughout the intervention, the participants will have a continuous support from community volunteers that act as program facilitators. Facilitators will consult the professional health providers if any problems arise during the intervention sessions. Participants will also be referred to relevant health providers if the problem could not be handled by the program facilitators.

Table 4: The frequency of lifestyle modification intervention sessions and delivery

**Study period and frequency of sessions**	**Total sessions**	**Deliver by**
**0 to 3rd month:**<br>Intensive intervention<br>2 weekly sessions for 3 months	6	Professionals (Doctors, Nurse, Dietician, Physical trainer, Diabetic Educator or Community Health Nurse)
**4th to 6 month:**<br>Less-intensive intervention<br>1 monthly sessions for 3 months	3	Facilitators with supervision by professionals
**7th to 9th month:**<br>Maintenance of intervention<br>1 monthly sessions for 3 months	3	Facilitators with supervision by professionals
**10th to 12th month:**<br>Maintenance of intervention	0	Follow up of participants by facilitators via phone, e-mail or home visits
**Total sessions**	12	

All intervention sessions will be conducted either in groups of 20 people or smaller groups of (5 – 8) people depending on the type of activities (i.e. lecture, seminar, group work or discussion).

Table 5 provides detailed information on session titles, frequency, content focus and the Health Belief Model constructs.

Table 5: The Community-based Healthy Lifestyle Intervention Program (Co-HELP)

Health Belief Model Construct	Session and title	Content and purpose of the session
Perceived susceptibility	**Session 1**<br><br>Introduction to Co-HELP.<br><br>Risk factors for Type 2 Diabetes	• Introduction to the program and other group members<br>• To increase knowledge about Diabetes and the risk factors.<br>• To understand the signs, symptoms, complications and management of chronic diseases.<br>• Emphasis on benefits of lifestyle modification; diet, physical activity, smoking & alcohol cessation.
Perceived benefits	**Session 2**<br><br>Healthy eating	• To increase knowledge about healthy eating, principles of healthy diet, food pyramid & portion size<br>• Improving quality of food choice with tips to reduce salt, sugar and fatty intake,<br>• What is 'healthy plate model'& 'snacks food"<br>• Diseases related to unhealthy diet.<br>• Pre & Post test questionnaire.
Perceived benefits	**Session 3**<br><br>Guide to exercise and physical activity	• What is exercise and physical activity?<br>• Concept of energy balance, diet and exercise.<br>• Making preparation to exercise, types and different phases of exercise.<br>• For participants to know the appropriate types of exercise for middle aged and weight reduction purposes.<br>• Common barriers to exercise and coping strategies.<br>• Counting the heart rate and goal setting.
Perceived barriers	**Session 4**<br><br>Keys to healthy eating and groceries shopping	• Identify obstacles, learn on problem solving and decision making.<br>• Tips to improve the quality of food choices & shopping tips i.e; reading food labels and nutritional facts, preparation of shopping lists and plan ahead.<br>• To know what types of food rich in salt, sugar and fats.

		• What are sweeteners?<br>• Tips on menu choices while eating in outside i.e; restaurant or food stall.<br>• Good or bad choice of specific dishes according to different ethnic groups.
Perceived barriers	**Session 5**<br><br>Guide to lose weight and healthy cooking method	• Introduction to overweight and obesity problems.<br>• Measuring your own weight, BMI, waist circumference, BP and Blood Sugar.<br>• Knowing your ideal body weight and goal setting for weight reduction.<br>• Health benefits of achieving the targeted goals and energy balance.
Cues to action	**Session 6**<br><br>Exercise-10,000 steps	• Outdoor activity at the recreational park.<br>• Group walking in the community.
Cues to action	**Session 7**<br><br>Dietary interactive session	• Interactive session with participants, divided in smaller groups of 5- 8 people<br>• Problem solving, role play and group presentations.<br>• To recap and feedback of previous dietary modules.
Cues to action	**Session 8**<br><br>Exercise- Indoor exercise and during leisure time	• Interactive session with participants<br>• Flexibility and resistance exercise using elastic bands<br>• Aerobic exercise
Cues to action	**Session 9**<br><br>Creating a healthier recipe, cooking demonstration and healthy meal contest	• Interactive session with participants<br>• Practical meal planning and preparation,<br>• How to modify 2 famous local recipes of Negeri Sembilan into healthier recipes.<br>• Cooking demonstration by community-volunteer.<br>• Tips to reduce consumption of saturated fat (especially from coconut milk) in local dishes.<br>• Conducted a 'healthy meal contest' by modification of recipes
Self-efficacy	**Session 10**<br><br>Strengthen you exercise programs	• Outdoor aerobic exercise.<br>• Keep active and prevent from injury.<br>• How to achieve 150 minutes per week and moderate intensity exercise.
Self-efficacy	**Session 11**	• How to maintain your weight during Ramadhan (fasting month) and Syawal (Eid celebration)

	Motivational talk and sharing of experience	• Sharing of personal experienced by 2 role models (a retired government servant and a diabetic patient)<br>• Encourage other participants to share their own experiences and motivations about staying healthy.
Self-efficacy	**Session 12**<br><br>Looking back and looking forward	• Looking backward is to flash back on all the activities that have been conducted and participated.<br>• Looking forward is how to keep motivated and sustained healthy lifestyle.<br>• All participants signed up 'My personal Lifestyle Contract'.<br>• Token of appreciation to all participants<br>• Selection of best male and best female participants whom achieved the most weight loss, full attendance in group sessions and manage to reverse to normal blood glucose level.

Participants from the intervention group are also encouraged to self-monitor their body weight, biochemical results, diet and physical activity. A specific diary will be given for participants record on their progress. In addition, they will also receive handouts, pamphlets and booklets of various health issues. Pre-tests and post-tests will be conducted after each educational session as to evaluate their understanding of the topics discussed earlier. Information gathered during initial appointment with dietitian will be used to design participants' diet plan, short term and long term weight loss goals and behaviour modification. The dietary advice given by the dietitian was based on the Malaysian Nutritional Guidelines [54-56]. Each participant's daily recommended energy requirement was estimated by the dietitian based on 3-day food records. The participants are encourage to aim at 5-10% weight loss at 6-month and continue to lose weight up to 12 months. Participants are also encouraged to increase their physical activity to a minimum of 150 minutes per week and aimed for moderate intensity exercises. A six-kilometer walking track will be set up within the community area. The pathway chosen for walking activity need to be far away from the main road to ensure safety. At every kilometer distance, a banner will be put up with motivational health

messages. Participants are encouraged to form their own walking groups and use pedometers to assess their progress.

iii) Control group

Participants in the usual care group receive standard health education from primary care providers in the clinic. In addition, they also provide with pamphlets and booklets about various health topics. A specific diary is also given for them to record their weight, diet and physical activities and other blood test results.

Table 6: Summary of differences between lifestyle modification intervention and conventional intervention groups

	**Lifestyle modification intervention group**	**Usual care group**
Goals Setting		
- Weight loss	5-10% of initial weight	5-10% of initial weight
- Physical activity	150 minutes/week	150 minutes/week
- Reduce calorie diet	20-25 kcal/kg	20-25 kcal/kg
- Behaviour change	Smoking & alcohol avoidance	Smoking & alcohol avoidance
Intervention format	Structured<br>Group sessions	Unstructured
Frequency of follow up	Total of 12 sessions	Depends on the primary care clinic follow up
Activities	Will receive healthy lifestyle modification intervention in group base activities like -<br>Lectures<br>Group works/ discussion<br>Aerobic exercise<br>Healthy food choice & cooking demonstration<br>Behaviour modification tips	Will receive healthy lifestyle information and education materials

6.3 Measurements

i) Anthropometry and blood pressure

Clinical measurements will include weight, height, BMI, waist circumference and blood pressure, taken by trained personnel using appropriate calibrated measuring tools. The participants will be instructed to wear light clothing and without shoes during measurements. Height and weight will be taken using a stadiometer and SECA digital scale, to the nearest 0.1 cm and 0.1 kg respectively and these measurements will be used to calculate Body Mass Index (BMI). BMI is calculated using the formula of weight in kg divided by height in m^2 (kg/m^2). In this study, the BMI is according to the revised cut-offs for Asian classification [57]. Patients with BMI between 23.0 kg/m^2 to <27.5 kg/m^2 is classified as overweight and a BMI of $\geq$ 27.5 kg/m^2 is classified as obese. Waist circumference will be taken to nearest 0.1 cm using SECA measuring tape and according to the WHO procedure (1995) [58]. Blood pressure will be measured with an automatic digital blood pressure monitor (OMRON HEM -907 model). The participants are required to be seated while two measurements are taken for average value after resting for at least 10 to 15 minutes.

ii) Biochemical

All participants will be required to fast overnight prior to each blood test for a minimum of eight hours. Plasma levels of fasting glucose and 2 hour post 75 g glucose load are assayed using the GLUC method for the Dimension Clinical Chemistry System (by Siemens, Newark, U.S.A). To minimize the influence of any acute incidental reaction, participants will be advised not to engage with strenuous physical activity for at least 12 hours before blood sampling. The Bio-Rad D-10 Hemoglobin A1C program system will be used for the percentage determination of HbA1C in human whole blood using ion-exchange high performance liquid chromatography method (HPLC).

The total cholesterol (TC), high density lipoprotein (HDL) and triglyceride (TG) are analyzed using the CHOL, AHDL and TGL method for the Dimension Clinical Chemistry System (by Siemens, Newark, U.S.A). Whereas, low density lipoprotein (LDL) is calculated using the Friedewald equation [59].

iii) Physical activity

Physical activity (PA) will be measured using the short form International Physical Activity Questionnaire (IPAQ). The IPAQ was translated and validated in different languages including the Malay version of IPAQ [60]. Participants' weekly MET-minutes of walking, moderate and high intensity activity will be summed up and categorized into low physical activity (i.e., if < 600 MET-minutes/ week), moderate physical activity (i.e., if 600 -1500 MET-minutes/ week) and high physical activity (i.e., if ≥ 1500 MET-minutes/ week) [61]. The dietary intake will be measured using the 24 hours diet recall. Participants' are asked to record their dietary intake for three days (two week-days and one week-end) and the average measurement will be taken.

iv) Health-related quality of life

The health-related quality of life (HRQOL) will be assessed using a bilingual version of short-form health survey (SF-36) questionnaire. The SF-36 was translated and validated in Malaysia [62] and the Malay version of SF-36 has shown to be reliable and valid with Cronbach alpha coefficient for all subscales exceeded the recommended 0.70 [63]. It contains 36 items which measures eight health domains; physical functioning (PF), role-physical (RF), bodily pain (BP), general health (GH), vitality (VT), social functioning (SF), role-emotion (RE) and mental health (MH)[64]. The eight domains will be scored from 0 to 100 indicating worst to best possible health.

All the scores will further summarized into the Physical Component Summary score (PCS) and Mental Component Summary score (MCS).

6.4 Outcome measures

The primary outcome measures for this study are fasting blood glucose, 2-hour plasma glucose and HbA1C. Secondary outcome measures are weight, BMI, waist circumference, Total cholesterol, Triglyceride, LDL cholesterol, HDL cholesterol, Systolic and Diastolic blood pressure, physical activity, diet intake and HRQOL. All these will be taken at baseline, six months and twelve months except for HRQOL that will be measured at baseline and twelve months. The primary assessment time-point of this study is at 6-month to measure the immediate effect of the intervention. The final visit which occurred at 12-month will be used to measure sustainability of the intervention.

7.0 HOW TO ENSURE GOOD RESPONSE RATE?

i) Good response rate from the participants can be achieve by conducting regular meeting with the study partners to discuss about the progress and problems encounter. Immediate action will be taken if any problems arise.

ii) Community volunteers will play their role in terms of social visits and follow up for participant failed to turn up.

iii) To prescribe lifestyle modification interventions that are tailored with the Malaysian lifestyle and culture.

iv) To try our best to arrange the intervention activities during weekend and flexible with the participant’s time

8.0 STUDY VARIABLES

Independent variables	Dependent variables
i. Age	a) Changes in Weight (kg)
ii. Gender	b) Changes in Body Mass Index (kg/m^2)
iii. Ethnic group	c) Changes in waist circumference (cm)
iv. Education level	a) Changes in fasting plasma glucose (mmol/L), 2hPG (mmol/L) and HbA1C %
v. Socio-economic status	
vi. Family history of Diabetes	b) Changes in TC, HDL, LDL, TG (mmol/L)
vii. History of smoking	c) Change in SBP and DBP (mmHg)
viii. History of alcohol intake	

9.0 STUDY MEASUREMENTS AND INSTRUMENTS

Measurements	Instruments	Quality
Physical activity	Physical Activity Readiness Questionnaire (PAR-Q) International Physical Activity Questionnaire (IPAQ – short form)	Validated
Diet	24 hr diet recall	Validated
Weight	Electronic weighing machine	Calibrated
Height	Stadiometer	Standardized
Waist circumference	Measuring tape	Standardized
Blood Glucose	Laboratory analysis of serum plasma glucose	Calibrated
Lipid Profile	Laboratory analysis of serum lipid	Calibrated
Blood pressure	Digital blood pressure	Calibrated

10.0 DATA COLLECTION

The data collection for both groups will be done at the same interval:

i) 1st data collection : At baseline

ii) 2nd data collection: After 6 months

iii) 3rd data collection: After 12 months

Table 8: Summary of data collection and time interval

Measurements	Screening	Baseline	6-month	12-month
**Questionnaire**				
Socio-demography	X			
PAR-Q	X			
IPAQ-Short form		X	X	X
24-hour diet recall		X	X	X
SF-36 HRQOL		X		X
**Anthropometry**				
Weight	X	X	X	X
Height	X	X	X	X
BMI	X	X	X	X
Waist circumference	X	X	X	X
**Clinical**				
Blood Pressure	X	X	X	X
**Biochem**				
RBS	X			
FPG		X	X	X
2-hPG		X	X	X
HbA1C		X	X	X
Total cholesterol		X	X	X
HDL cholesterol		X	X	X
Triglycerides		X	X	X

11.0 DATA PROCESSING

Raw data obtained from the study will be entered into the Statistical Package for Social Sciences (SPSS) software version 19.0. The data will be clean and check for double entry or outliers before performing the analysis. The data input by the researcher.

12.0 DATA ANALYSIS

Patient demographic, clinical variables and baseline value will be compare between groups by the use of independent t-test for normally distributed variables and Mann Whitney test for non-normally distributed variables. Chi-square test will be use for discrete variables. The analysis will be done according to the intention-to-treat (ITT) principle. The missing values will be replaced by

the last readings carried forward. General Linear Model repeated measures ANOVA will be conducted to examine the changes overtime within-groups and between-groups. In the repeated measures model, the baseline value of co-morbidities, medication used, smoking and alcohol consumption were used as covariates in analysis for lipid profiles and blood pressure measurements. The outcome results from baseline to follow up at 6 months and 12 months will be presented as mean group differences, 95% confidence interval, p value and partial eta squared. A p-value of <0.05 was considered statistical significant and effect size as proposed by Cohen (1998) were (0.01= small effect, 0.06= moderate effect and 0.14= large effect [65]. All statistical analysis will be performed using the SPSS software version 19.0 (SPSS Inc, 2009, Chicago, Illinois)

13.0 STUDY LIMITATIONS AND MEASURES TO MINIMIZE IT

i) Measurement bias can occur while taking recording or taking measurements (i.e.; recording the weight, height, Blood Pressure, waist circumference). The inter-observer variation can be avoided by training of the staffs conducting the measurements and using same type of instruments. The machines or instruments must be calibrated before use.

ii) Information bias on physical activity and dietary intake recall. This can occur whereby the respondents might not give the true or exact answers to the questions, either they have forgotten or refused to reveal some information to others. Sometimes, the interpretation of questions might be differently interpreted by the respondents. The limitation unfortunately has to be acknowledged.

iii) Drop out of participants from the study can occur at any time and without reasons. Therefore, measures have to be taken by conducting regular meeting with the partner organizations to discuss about progress and problems encountered. Immediate action should be taken if any

problems arise. The peer-educator formed among the MDA members can help to track the defaulter cases by means of social visit and follow up.

14.0 ETHICAL CONSIDERATION

A written consent will be obtained from the patient before any intervention or procedure to be conducted on them. The patient will be informed and explained about the purpose of the study. The patient has the right to refuse or disagree to participate in the study. Participants' safety is the prime concern in this study. The risk to participants in this study is modest primarily when related to physical activity. Adoption of vigorous physical activity regimen can increase the risk of cardiovascular events in previously sedentary individuals; hence this study will focus on adoption of light to moderate physical activity program such as brisk walking and aerobic exercises. The participants will be screened for physical capacity using Physical Activity Readiness Questionnaire (PAR-Q) and will be referred to relevant specialist if required. Participants will be educated about the sign and symptoms of angina and heart attack that they should inform immediately. All potential adverse events of intervention specific to physical exercise, injuries and orthopedic conditions will be recorded, monitored and reported to the Medical Ethics Committee. Ethics clearance will be obtained from the Medical Ethics Committee of UMMC.

15.0 PUBLIC HEALTH IMPACT

i) Only a small percentage of Malaysian adults with pre-diabetes are aware that they are at high risk of developing type 2 diabetes. Not many made any lifestyle changes as to reduce the diabetes risk.

ii) Lifestyle modification has been shown effective in reducing the risk of diabetes in randomized control trials. However, the main challenge is to translate this evidence into a routine community setting and provide a feasible effective intervention.

iii) There should be a concerted effort all round including smart partnership between the government agencies, Non-governmental agencies (NGOs) and research institutions to join forces to curb this problem holistically.

16.0 GANTT CHART

**Activities :**	**Year 2011**											
	Jan	Feb	Mar	Apr	May	Jun	Jul	Aug	Sep	Oct	Nov	Dec
1. Preparation of study proposal												
2. Submission to ethical review board												
3. Protocol approval from UMMC ethical board												
4. Submission to IPPP for post-graduate research grant												
5. Preparation of study materials												
6. Health screening and recruitments												
7. Training of the trainers												
8. Randomization & Baseline data collection												
9. Lifestyle interventions												
**Activities :**	**Year 2012**											
	Jan	Feb	Mar	Apr	May	Jun	Jul	Aug	Sep	Oct	Nov	Dec
10. Lifestyle interventions												
**Activities :**	**Year 2013**											
	Jan	Feb	Mar	Apr	May	Jun	Jul	Aug	Sep	Oct	Nov	Dec
11. Data collection												
12. Data collection												
13. Data collection												
14. Data collection												
**Activities :**	**Year 2014**											
	Jan	Feb	Mar	Apr	May	Jun	Jul	Aug	Sep	Oct	Nov	Dec

15. Data analysis												
16. Final report												

17.0 References

1. King, H.R., M Global estimates for prevalence of diabetes mellitus and impaired glucose tolerance in adults. WHO Ad Hoc Diabetes Reporting Group, Diabetes Care, 1993. Vol 16(No 1): p. 157-177.
2. International Diabetes Federation, Global estimates of the prevalence of diabetes for 2010 and 2030. Diabetes Atlas, 2009.
3. Zaini, A., Where is Malaysia in the midst of the Asian epidemic of Diabetes Mellitus? Diabetes Research and Clinical Practice, 2000. 50(2): p. S23-S28.
4. Institute of Public Health, M.o.H.M., Diabetes Mellitus Group. The Third National Health and Morbidity Survey 2006 (NHMS III), 2008.
5. Institute of Public Health, Diabetes Mellitus Group. The third National Health and Morbidity Survey (NHMS III), Ministry of Health Malaysia, 2006.
6. Knowler WC, B.-C.E., Fowler SE, Hamman RF, Lachin JM, Walker EA, Nathan DM; Diabetes Prevention Program Research Group, Reduction in the incidence of type 2 diabetes with lifestyle intervention or metformin. The New England Journal of Medicine, 2002. 346(6): p. 393-403.
7. Tuomilehto J, L.J., Eriksson JG, Valle TT, Hämäläinen H, Ilanne-Parikka P, Keinänen-Kiukaanniemi S, Laakso M, Louheranta A, Rastas M, Salminen V, Uusitupa M; Finnish Diabetes Prevention Study Group., Prevention of type 2 diabetes by changes in lifestyle among subjects with impaired glucose tolerance. The New England Journal of Medicine, 2001. 344: p. 1343-1350.
8. Ambady Ramachandran et al, Diabetes in Asia. The Lancet, 2010. 375: p. 408-417.
9. H. King , R.A., W.Herman Global burden of diabetes, 1995-2025: prevalence, numerical estimates and projections. Diabetes Care, 1998. 21(9): p. 1414-1431.
10. (IDF), I.D.F., Executive Summary, 2nd edition. Diabetes Atlas, 2003.
11. Wild S, R.G., Green A, Sicree R, King G., Global prevalence of diabetes. Estimation for the year 2000 and projections for 2030. Diabetes Care, 2004. 27: p. 1047-1052.
12. J.E.Shaw, R.A.S., P.Z.Zimmet, Global estimates of the prevalence of diabetes for 2010 and 2030. Diabetes Atlas. Diabetes Research and Clinical Practice, 2009. 87: p. 4-14.
13. McCarthy D, Z.P., Diabetes 1998 to 2010: Global estimates and projections. Interact in International Diabetes, Melbourne, Australia, 1997. ISBN064020.2448.10.
14. Yoon KH, L.J., Kim JW, et al, Epidemic obesity and type 2 diabetes in Asia. The Lancet, 2006. 368: p. 1681-1688.
15. Ng MC, L.S., Ko GTC, et al, Familial early onset type 2 diabetes in Chinese: the more signifiacnt roles of obesity and genetics tahn autoimmunity. Diabetes Care, 2001. 24: p. 667-671.
16. Groop L, The molecular genetics of non-insulin dependent diabetes mellitus. Journal of Internal Medicine, 1997. 241: p. 95-110.

17. Tan, J.T., et al., A family history of type 2 diabetes is associated with glucose intolerance and obesity-related traits with evidence of excess maternal transmission for obesity-related traits in a South East Asian population. Diabetes Research and Clinical Practice, 2008. 82(2): p. 268-275.
18. UK Prospective Diabetes Study Group: UK Prospective Study 16, Overview of 6 years' therapy of type 2 diabetes : A progressive disease Diabetes Journal, 1995. 44: p. 1249-1258.
19. American Diabetes Association (ADA), Diagnosis and classification of diabetes mellitus. Diabetes Care, 2008. 31: p. S55-S60.
20. Ronal T. Ackermann, E.A.F., Edward Brizendine, Honghong Zhou, David G.Marero, Translating the Diabetes Prevention Program into the Community. THe DEPLOY Pilot Study. American Journal Preventive Medicine, 2008. 35(4): p. 357-363.
21. de VF, D.J., Jager A, et al, Relation of impaired fasting and postload glucose with incident of type 2 diabetes in a Dutch population: the Hoorn study. JAMA, 2001. 285: p. 2109-2113.
22. Wong MH , G.K., Heng D, Chew SK, Chew LS, Tai ES et al, The Singapore impared glucose tolerance follow-up study : Does the ticking clock go backward as well as forward? Diabetes Care, 2003. 26: p. 3024-3030.
23. Chou P, L.C., Wu GS, Tsa ST et al, Progression of type 2 diabetes among high-risk group in Kin-Chen, Kinmen: Exploring the natural history of type 2 diabetes. Diabetes Care, 1998. 21: p. 1183-1187.
24. Pan XR, L.G., Hu YH, Wang JX, An ZX, Hu ZX, Lin J, Xioa JZ, Cao HB, Liu PA, Jiang XG, Jiang YY, Wang JP, Zheng H, Zhang H, Bennett PH, Howard BV et al, Effects of diet and exercise in preventing NIDDM in people with impaired glucose tolerance: the Da Qing IGT and Diabetes Study. Diabetes Care, 1997. 20: p. 537-544.
25. Kosaka K, N.M., Kuzuya T, Prevention of type 2 diabetes by lifestyle intervention : a Japanese trial in IGT males. Diabetes Research and Clinical Practice, 2005. 67: p. 152-62.
26. Ramachandran A, S.C., Mary S, Muksh B, Bhaskar A, Vijay V, The Indian diabetes prevention programme shows that lifestyle modification and metformin prevent type 2 diabetes in Asian Indian with impaired glucose tolerance (IDPP-1). Diabetologia, 2006. 49: p. 289-297.
27. Kazue Yamaoka, T.T., Efficacy of lifestyle education to prevent type 2 diabetes : A meta-analysis of randomized controlled trials. Diabetes Care, 2005. 28(11): p. 2780-2786.
28. Rampal, L., et al., Prevalence, awareness, treatment and control of hypertension in Malaysia: A national study of 16,440 subjects. Public Health, 2008. 122(1): p. 11-18.
29. Rampal S, R.L., Rahmat R, Azhar MZ, Yee GY, Mohamed M, Variation in the prevalence, awareness and control of diabetes in multiethnic population : a nationwide population study in Malaysia. Asia Pacific Journal Public Health, 2009. 22(2).
30. Osman, A., et al., Prevalence of NIDDM and Impaired Glucose Tolerance in Aborigines and Malays in Malaysia and their relationship to sociodemographic, health and nutritional status. Diabetes Care, 1993. 16: p. 68-75.
31. S.P.Chan, P.R., L.F.Lee, F.L. Hew, S.I.Ikram, M.Manisavagar, Y.Zaitun, A.S.Hazizi, A.C. Tee, A.S.M. Khir, C.T. Chua, S.F. Alhady, A.Zaini, Progression of glucose intolerance and diabetes mellitus in a 10 year follow up study of non-communicable diseases in a rural population. Presented at the 10th Congress of Asian Federation of Endocrine Societies Bangkok, 1999.
32. K.G.M. Alberti, P.Z., J.Shaw,, International Diabetes Federation : a concensus on type 2 diabetes prevention. Diabetic Medicine, 2007. 24: p. 451-463.

33. Department of Statistics Malaysia, Population distribution and basic demographic characteristics. 2010.
34. District council Seremban, http://seremban.ns.gov.my. Accesed on 1 September, 2013. 2010.
35. Shaharudin Idrus, A.S.H., Abdul Hadi Harman Shah, Ruslan Rainis, Neighbourhood Expansion and Urban Livability in Seremban Municipality Area, Malaysia. Malaysian Journal of Environmental Management 2010. 11(1).
36. Lindstrom J, T.J., The Diabetes Risk Score : a practical tool to predict type 2 diabetes risk. Diabetes Care, 2003. 26: p. 725-731.
37. American Diabetes Association (ADA), Diabetes Risk Test. 2007.
38. Aekplakorn W, B.P., Woodward M, Sritara P, Cheepudomwit S, Yamwong S, , A risk score predicting incident diabetes in the Thai population. Daibetes Care, 2006. 29: p. 1872-1877.
39. Griffin SJ, L.P., Hales CN, Kinmoth AL, Wareham NJ,, Diabetes risk score: towards earlier detection of type 2 diabetes in general practice. Diabetes Metab Res Rev, 2000. 16: p. 164-171.
40. Ministry of Health Malaysia, Management of Type 2 Diabetes Mellitus 4th edition. Clinical Practice Guideline. , 2009.
41. Li G, Z.P., Wang J, Greg EW, Yang W, Gong Q, et al, The long term effect of lifestyle intervention to prevent diabetes in China Da Qing Diabetes Prevention Study: a 20 year follow-up study. The Lancet, 2008. 371: p. 1783-1789.
42. Kulzer, B., et al., Prevention of diabetes self-management program (PREDIAS): effects on weight, metabolic risk factors, and behavioral outcomes. Diabetes Care, 2009. 32(7): p. 1143-6.
43. Absetz P, V.R., Oldenburg B, Heinonen H, Nissinen A, Fogelholm M, Ilvesmaki V, Tajla M, Uutela A, Type 2 diabetes prevention in the "Real World". Diabetes Care, 2007. 30: p. 2465-2470.
44. Ackermann RT, E.A.F., Edward Brizendine, Honghong Zhou, David G Marero, Translating the Diabetes Prevention Program into the Community. THe DEPLOY Pilot Study. American Journal Preventive Medicine, 2008. 35(4): p. 357-363.
45. Boltri JM, D.-S.Y., Seale JP, Shellenberger S, Okosun IS, Cornelius MS, Diabetes prevention in faith-based setting: results of translational research. J Public Health Manag Pract, 2008. 14: p. 29-32.
46. Jaber, L.A., et al., Feasibility of group lifestyle intervention for diabetes prevention in Arab Americans. Diabetes Research and Clinical Practice, 2011. 91(3): p. 307-315.
47. Jeffery A. Katula, M.Z.V., Erica L. Rosenberg, Caroline S. Blackwell, Timothy M. Morgan, Michael S. Lawlor, David C. Goff Jr., One-year resulrs of a community-based translation of the diabetes prevention program. Healthy-living partnerships to prevent diabetes (HELP PD) project. Diabetes Care, 2011. 34: p. 1451-1457.
48. Norma S, Z.M., Azmi MT, Nor Aryana H, The effectiveness of lifestyle intervention among pre-diabetes patients in Melaka, Malaysia. Journal of Nursing and Health Science (IOSR-JNHS), 2013. 2(1): p. 14-21.
49. Ismail IS, W.M.W.B., Mohd Ismail Noor, Nor Azmi Kamaruddin, Rabindarjeet Singh, Noor Hisham Abdullah, Zanariah Hussein, Fuziah Md Zain, Lee Lai Fun, Siti Hawa Mohd Taib,, Management of Obesity. Clinical Practice Guidelines by Ministry of Health Malaysia, 2004. 1: p. 1-58.
50. Ministry of Health Malaysia, Clinical Practice Guidelines Management of Hypertension 3rd edition. 2008.

51. Diabetes Prevention Program research team, Group Lifestyle Balance University of Pittsburgh, United States, 2004, 2008, 2010, 2011.
52. State Health Department of Negeri Sembilan and Ministry of Health Malaysia, Integrated Educational Module for Non-Communicable Diseases. 2009. 1 and 2.
53. Rosenstock IM, S.V., Becker MH., Social learning theory and the Health Belief Model. Health Educ Q, 1988. 15(2): p. 175-183.
54. Malaysian Dietitians' Association, Medical Nutritional Therapy Guidelines for Type 2 Diabetes. Ministry of Health Malaysia, 2013.
55. National Coordinating Committee on Food and Nutrition, Malaysian Dietary Guidelines. Nutrition Division, Ministry of Health Malaysia, 2010.
56. Malaysian Dietitians' Association, Medical Nutritional Therapy for Type 2 Diabetes. Ministry of Health Malaysia, 2005.
57. WHO expert consultation, Appropriate body-mass index for Asian populations and its implications for policy and intervention strategies. Lancet, 2004. 363: p. 157-163.
58. Oka, R., et al., Comparison of waist circumference with body mass index for predicting abdominal adipose tissue. Diabetes Research and Clinical Practice, 2009. 83(1): p. 100-105.
59. Friedwald W.T, L.R., Fredrickson DS,, Estimation of the concentration of low-density lipoprotein cholesterol in plasma, without use of the preparative ultracentrifuge. Clin. Chem, 1972: p. 499-502.
60. Cora L. Craig, A.L.M., Michael Sjostrom,Adrian E. Bauman, Michael L. Booth,, International Physical Activity Questionnaire: 12 country reliability and validity. Med. Sci. Sports Exerc., 2003. 35(8): p. 1381-1395.
61. IPAQ Research Commitee, Guidelines for Data Processing and Analysis of the International Physical Activity Questionnaire (IPAQ)- Short and Long Forms. Revised November, 2005: p. 1-15; www.ipaq.ki.se.
62. Azman A.B., S.S., B. Rugayah, L.L. Low, A.A. Azian, S.Geeta, C.T. Tiew,, Quality of life of the Malaysian general population : results from a postal survey using the SF-36. Med J. Malaysia, 2003. 58: p. 694-711.
63. Sararaks S., A.A.B., L.L. Low, B. Rugayah, A.M. Aziah,, Validity and reliability of the SF-36: the Malaysian context. Med J. Malaysia, 2005. 60: p. 163-179.
64. Ware J.E. and Sherbourne C.D., The MOS 36-item short-form health survey (SF-36). Conceptual framework and item selection. Med Care, 1992. 30: p. 473-83.
65. Cohen J, Statistical power analysis for the Behavioural Sciences. Second edition. Hillsdale, New Jersey, 1998.
